# A curated global dataset of naturally occurring aphid-fungal associations

**DOI:** 10.1038/s41597-026-07691-5

**Published:** 2026-07-01

**Authors:** Ibtissem Ben Fekih, Gábor Pozsgai, Jørgen Eilenberg, Annette Bruun Jensen, Malgorzata Ruszkiewicz-Michalska, Kris AG Wyckhuys, Markus V. Kohnen, Said Amrani, Siegfried Keller, Ikbal Chaieb, Jiaxin Dai, Francisco Javier Sánchez-García, Sohaib H. Mazhar, Wenxiang Chen, Mark Goettel, Gábor L. Lövei, Minsheng You, Shijun You, Christopher Rensing

**Affiliations:** 1 Institute of Environmental Microbiology, College of Resources and Environment, Fujian Agriculture and Forestry University, Fuzhou, 350002 China; 2 Functional and Evolutionary Entomology, Gembloux Agro-Bio Tech, University of Liège, Passage des Déportés 2, 5030 Gembloux, Belgium; 3 State Key Laboratory of Agriculture and Forestry Biosecurity, Institute of Applied Ecology, Fujian Agriculture and Forestry University, Fuzhou, 350002 China; 4 Center of Applied Economic Studies of the Atlantic, University of the Azores, Angra do Heroismo, Terceira Portugal; 5 Department of Plant and Environmental Sciences, University of Copenhagen, Thorvaldsensvej 40, 1871 Frederiksberg C., Denmark; 6 Department of Algology and Mycology, Faculty of Biology and Environmental Protection, University of Lodz, Banacha 12/16, 90-237 Lodz, Poland; 7 Institute for Plant Protection, China Academy of Agricultural Sciences (CAAS), Beijing, China; 8 School of the Environment, University of Queensland, Saint Lucia, Australia; 9 Basic Forestry and Proteomics Research Center, College of Life Science, Fujian Provincial Key Laboratory of Haixia Applied Plant Systems Biology, Fujian Agriculture and Forestry University, Fuzhou, 350002 China; 10 Laboratoire de Biologie et de Physiologie des Organismes, Faculté des Sciences Biologiques, Université des Sciences et de la Technologie Houari Boumediène, BP 32 El Alia, Alger, 16111 Algeria; 11 Agroscope, Reckenholzstrasse 191, CH-8046 Zurich, Switzerland; 12 Laboratory of Production and Protection for a Sustainable Horticulture (LR21AGR03) Regional Centre of Research on Horticulture and Organic Agriculture, University of Sousse, 57, Chott Mariem, TN-4042 Tunisia; 13 Instituto Murciano de Investigación y Desarrollo Agrario y Alimentario (IMIDA), Murcia, Spain; 14 Institute of Sciences in Emergency Medicine, Department of Emergency Medicine, Guangdong Provincial People’s Hospital, Southern Medical University, Guangzhou, 510080 China; 15 Agriculture & Agri-Food Canada, Lethbridge, Canada; 16 Department of Agroecology, Aarhus University, Flakkebjerg Research Centre, DK-4200 Slagelse, Denmark; 17 HUN-REN UD Anthropocene Ecology Research Group, University of Debrecen, H-4032 Debrecen, Hungary

## Abstract

As key components of terrestrial ecosystems, fungi play vital roles in ecological processes and functions, and are associated with innumerable plant, vertebrate, and arthropod taxa. Among arthropod taxa, aphids (Hemiptera) are commonly found in both natural and agricultural ecosystems, where some species cause substantial crop damage. Here, we provide a novel and unique dataset, AphidFunga, compiling associations between fungi and aphids extracted from 412 scientific publications, spanning 167 years and covering 85 countries. Fungal and aphid taxonomies were revised to recent nomenclature, whereas association types were updated based on current knowledge. The AphidFunga database currently contains 2993 aphid-fungal association records, linking 365 aphid host taxa (species or genera) with 149 fungal taxa, 95% of which are entomopathogenic. The database is available in three formats: a combined comma-separated data table, a set of R data frames, and a MySQL relational database. The AphidFunga database lays the foundation for further research on fungus-mediated ecosystem processes and functions, and supports conservation science, policy development, and applications in crop protection and environmental management.

## Background & Summary

Fungi are among the world’s largest and most diverse groups of organisms^1,2^, with approximately 155,000 currently described species of an overall estimated 2-3 million fungal species^3–5^. Fungal mycelia permeate and enmesh soil, plant, and animal tissue and play essential roles in ecosystem health through diverse functions. Fungi interact with arthropods in various ways: 1) mutualistic, where both partners benefit by exchanging essential nutrients and protection^6–10^; 2) commensal, where one partner benefits without affecting the other^10,11^; 3) parasitic, where fungi live at the expense of their host, impairing its immune system and imposing fitness costs^12^, and 4) entomopathogenic, where fungi additionally cause an infectious disease which often leads to host mortality^12,13^. Besides these well-defined interactions, fungi with saprobic traits, as well as those commonly associated with plants or soil, are also found on the body surfaces or in the guts of arthropods, though these associations remain poorly understood^14,15^.

Previous checklists on entomopathogenic fungi, such as reports, monograph-type studies, and datasets, have primarily focused on entomopathogenic interactions at a regional or, in some cases, partially global scales^16–22^. These checklists are either outdated, geographically restricted, or exhibit narrow taxonomic coverage. Beyond entomopathogenicity, some studies have examined parasitic interactions, particularly between Laboulbeniomycetes (Ascomycota) and arthropods^23–25^. However, considerable gaps remain, with much yet to be explored, including host diversity, ecological and physiological impacts on hosts, and the evolutionary mechanisms underlying these associations^26,27^. Few databases have compiled a broader spectrum of arthropod-fungus associations, including non-pathogenic interactions. Pozsgai *et al*.^28^ presented the interactive CaraFun dataset documenting all known association types between carabids and fungi globally. Other studies have addressed ant-fungus associations^29,30^ and the intricate relationships between bark beetles and their symbiotic fungi^31,32^. Generally, insect-fungus associations can also be found in broad-spectrum interaction databases, such as Globi^33^, Mangal^34^, and Web of Life^35^.

Mapping this biostructure - the intricate associations between insects and fungi - is essential for understanding ecosystem processes and functions, and underpins both fundamental and applied research. Herbivorous arthropods cause substantial losses to global agriculture, threatening food security and rural livelihoods^36^, with these impacts predicted to worsen under climate change^37–39^. Aphids (Hemiptera: Aphidoidea) are serious pests of many important food, feed, and cash crops^40^. In 2025 Favret^41^ documented 6080 aphid species, assigned to 781 genera in 36 subfamilies, though these numbers are likely to increase. Of these, around 250 species are economically important^42^, causing direct losses by feeding on phloem sap and indirect damage by promoting sooty mould growth and/or transmitting plant pathogens^43^. Several groups of organisms contribute to conservation biological control of aphids, with entomopathogenic fungi among the most important^17^.

**Fig. 1:**
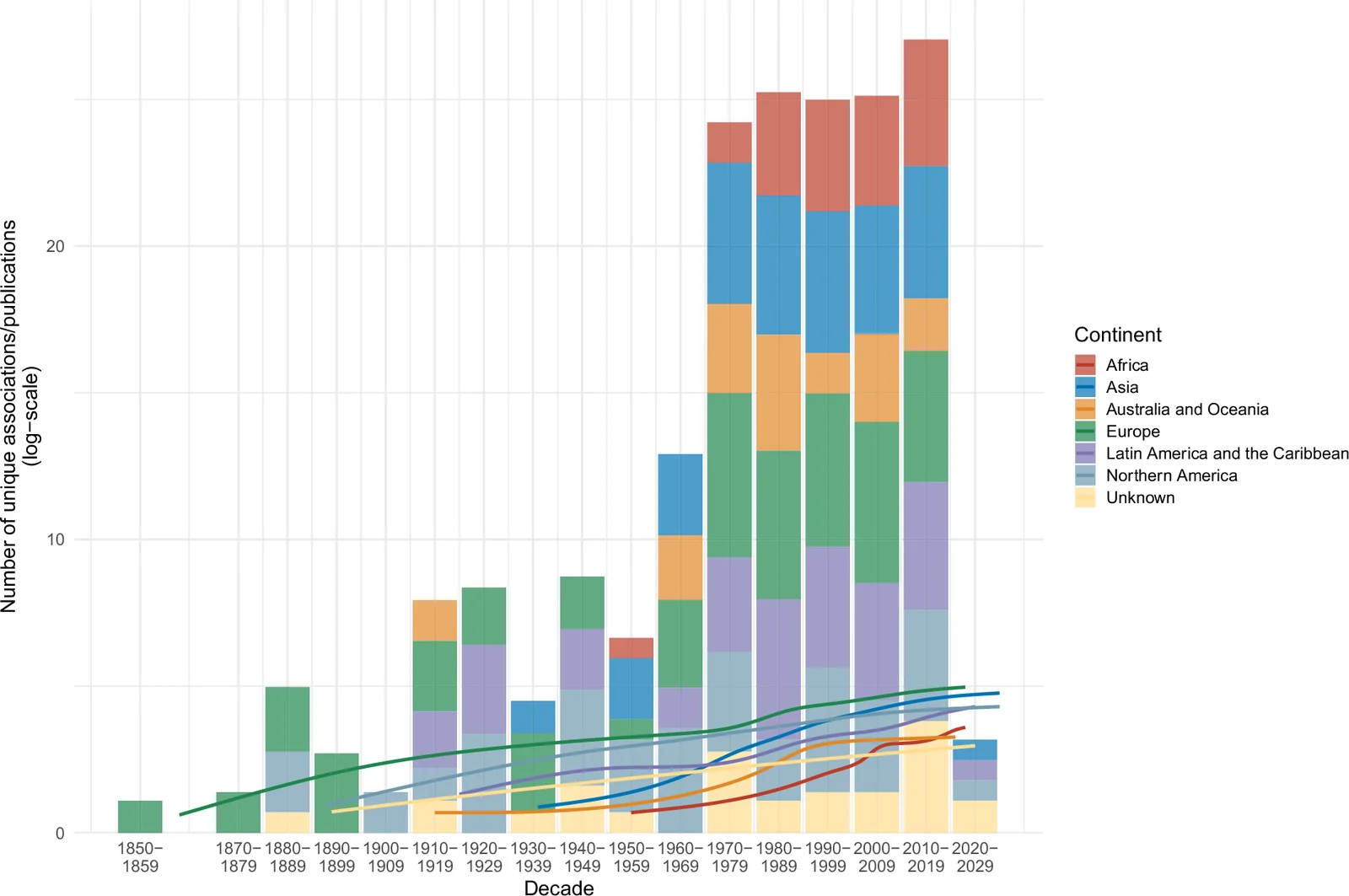
The number of reported unique associations (bars) and the number of unique papers reporting novel associations (smoothed lines) over time included in the AphidFunga database, separated by geographic regions.

Aphids host a great diversity of fungi, and of these associations/interactions pathogenicity is the most common^44^. Understanding these entomopathogenic interactions is key to assessing environmental integrity and enhancing ecosystem services, such as biological control of crop pests^45^. Despite their ecological and agricultural significance, a globally comprehensive dataset documenting aphid-fungal associations has been lacking. Here, we present AphidFunga that fills this critical gap by encompassing not only pathogenic interactions but also captures less-studied yet potentially significant associations, including those with saprobic, yeast-like, or plant pathogenic fungi. Documenting these cross-guild, non-pathogenic associations may help to elucidate how aphids contribute to fungal ecology. The database covers a wide breadth of knowledge, resulting from a thorough screening of over 800 scientific papers and books published between 1856 and 2025, with primary information extracted from 412 of these (Fig. 1).

**Fig. 2:**
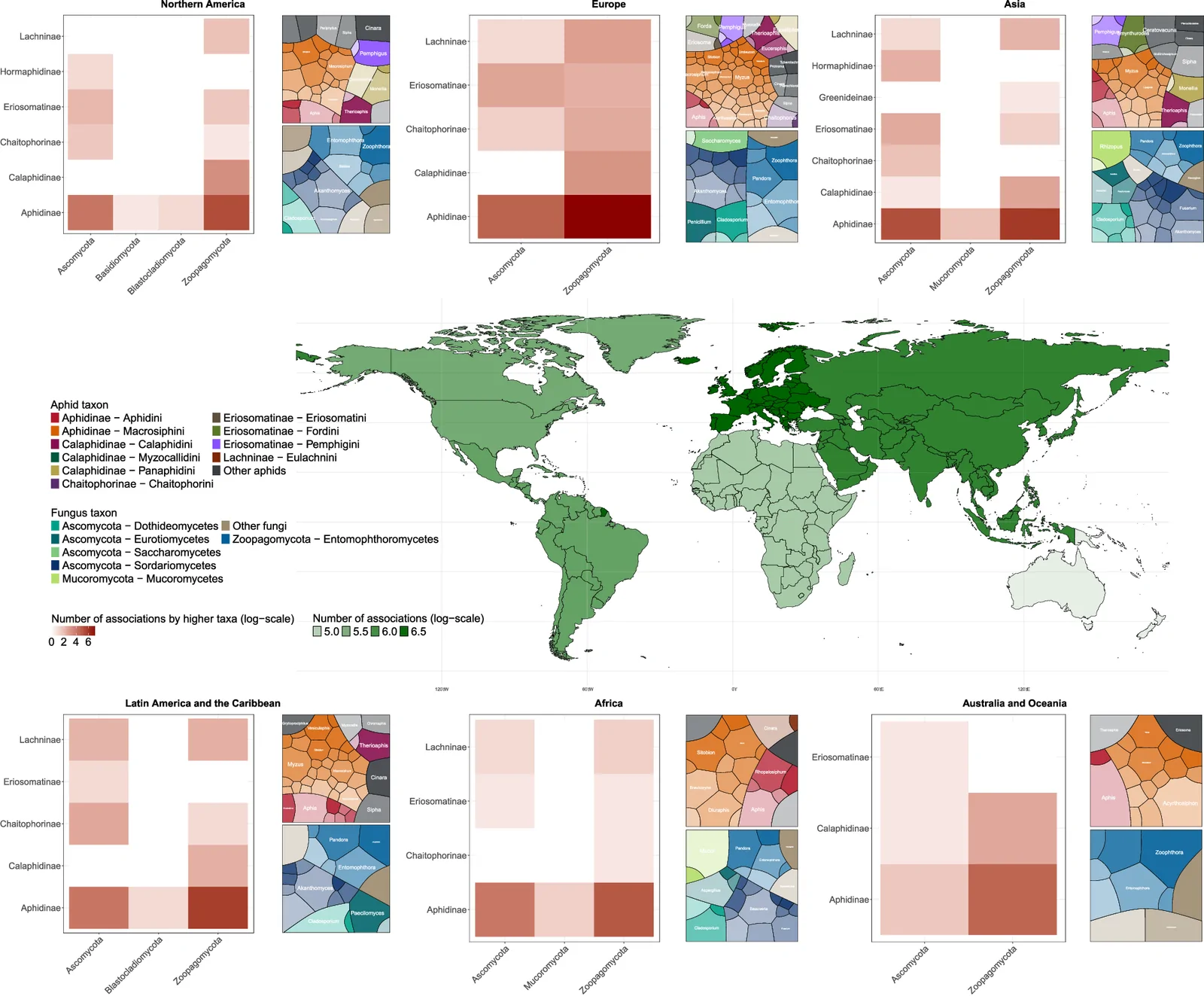
Data distribution across continents and taxa. Fill colour in the main map shows the number of recorded associations from each continent (log-scaled). Per continent, heatmaps show the frequency (log-scale) of associations among major fungal and aphid taxa, whereas paired Voronoi maps show the relative proportion of aphid and fungal taxa (top and bottom figures, respectively) in those associations.

**Fig. 3:**
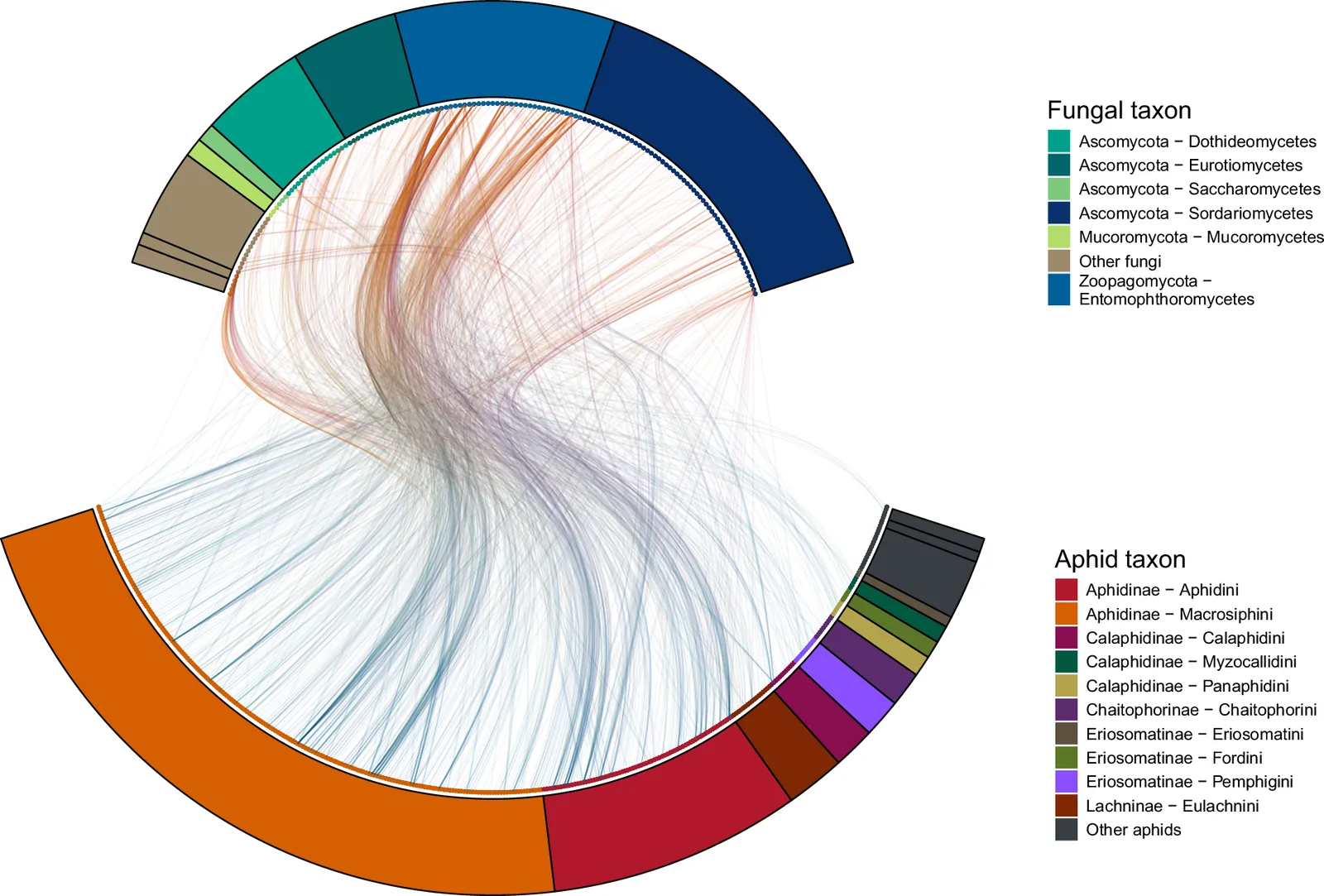
Bipartite network showing all associations between aphids and fungi, separated by major taxa. The length of a segment in the circle is proportional to the number of species the higher taxon encompasses. Each line represents an association between species or genera (when species are unidentified). Line color at the connecting point represents the major taxon of the associated partner.

AphidFunga is a unique open-access database that compiles 1524 distinct aphid-fungus associations (unique aphids-fungus taxon pairs), resulting in 2993 association records (geographically resolved occurrences of these associations, with records from one country listed only once, including flagged species see ‘*Data updates and formatting’* and ‘*Organizing data and decisions’*) from 85 countries (Fig. 2). The records cover 365 aphid species and genera (with species unidentified, see ‘*Organizing data and decisions’*), belonging to 14 subfamilies, 19 tribes, and 116 genera, as well as 149 fungal species and genera (with species unidentified), belonging to 5 phyla, 12 classes, and 56 genera (Fig. 3).

In our dataset, the fungal species with the broadest host ranges were *Entomophthora planchoniana* Cornu (164 aphid species), *Pandora neoaphidis* (Remaud. & Hennebert) Humber (163 species) and *Zoophthora aphidis* (H. Hoffm.) A. Batko (136 species). Among aphids, *Myzus persicae* (Sulzer, 1776) showed the most fungal associations, linked to 64 fungal species, followed by *Aphis gossypii* Glover, 1877 (35 associations) and *Aphis craccivora* Koch, 1854 (34 associations). Across countries, the most widely reported interactions were those between *P. neoaphidis* and *Acyrthosiphon pisum* (Harris, 1776) (26 countries), *Z. aphidis* and *M. persicae* (24 countries), and *P. neoaphidis* and *Sitobion avenae* (Fabricius, 1775) (23 countries).

The most numerous aphid subfamily was Aphidinae (272 species), which also carried the most fungal associates (131), but the ratio of fungal associate to aphid species was the lowest in this subfamily (0.48 – almost twice as much aphid species recorded than fungal associates linked to them). This ratio was the highest in Hormaphidinae where only 3 species were associated with 13 different fungi (ratio = 4.3).

**Fig. 4:**
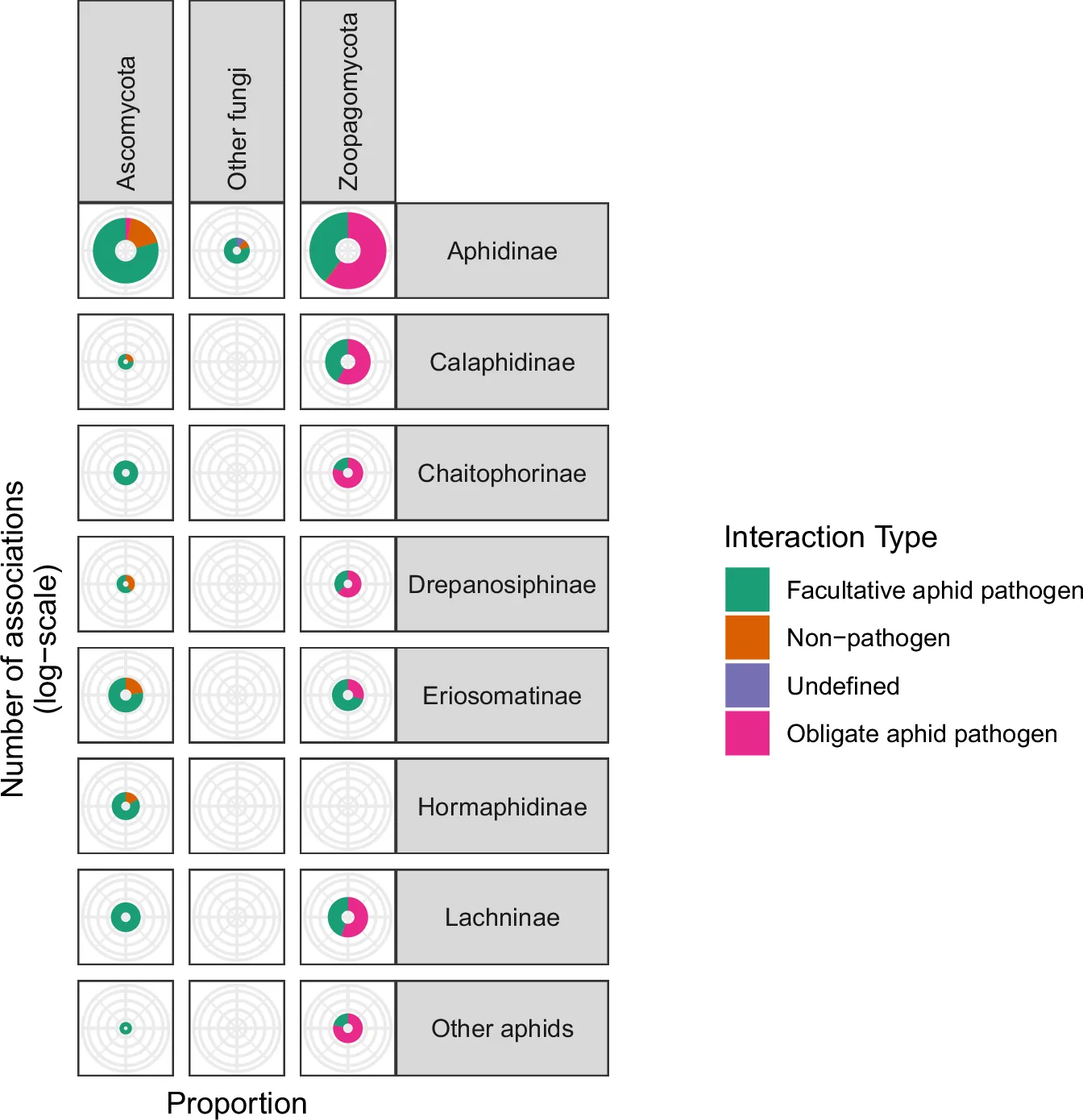
The proportion of interaction types between aphid subfamilies and fungal phyla. Fungal phyla and aphid subfamilies containing less than five species were grouped to “Other fungi” and “Other aphids”, respectively.

The diversity of pathogenic interactions varies across fungal phyla and continents, with most records in the Zoopagomycota (1094 interactions, ~76%), followed by Ascomycota (342 interaction, ~24%). Among the pathogenic interactions (~95%), those between members of the Aphidinae subfamily and fungi from the Zoopagomycota dominate (943 unique interactions, ~65%), with obligate aphid pathogens being the most prevalent (597 interactions, ~60%). Meanwhile, most fungal associations (all types) were recorded for aphids within the Aphidinae subfamily, with the highest proportion of obligate pathogenic interactions between fungi of the Zoopagomycota and aphids of the Chaitophorinae subfamily. Non-pathogenic associations were only recorded in fungi of the Ascomycota (Fig. 4).

**Fig. 5:**
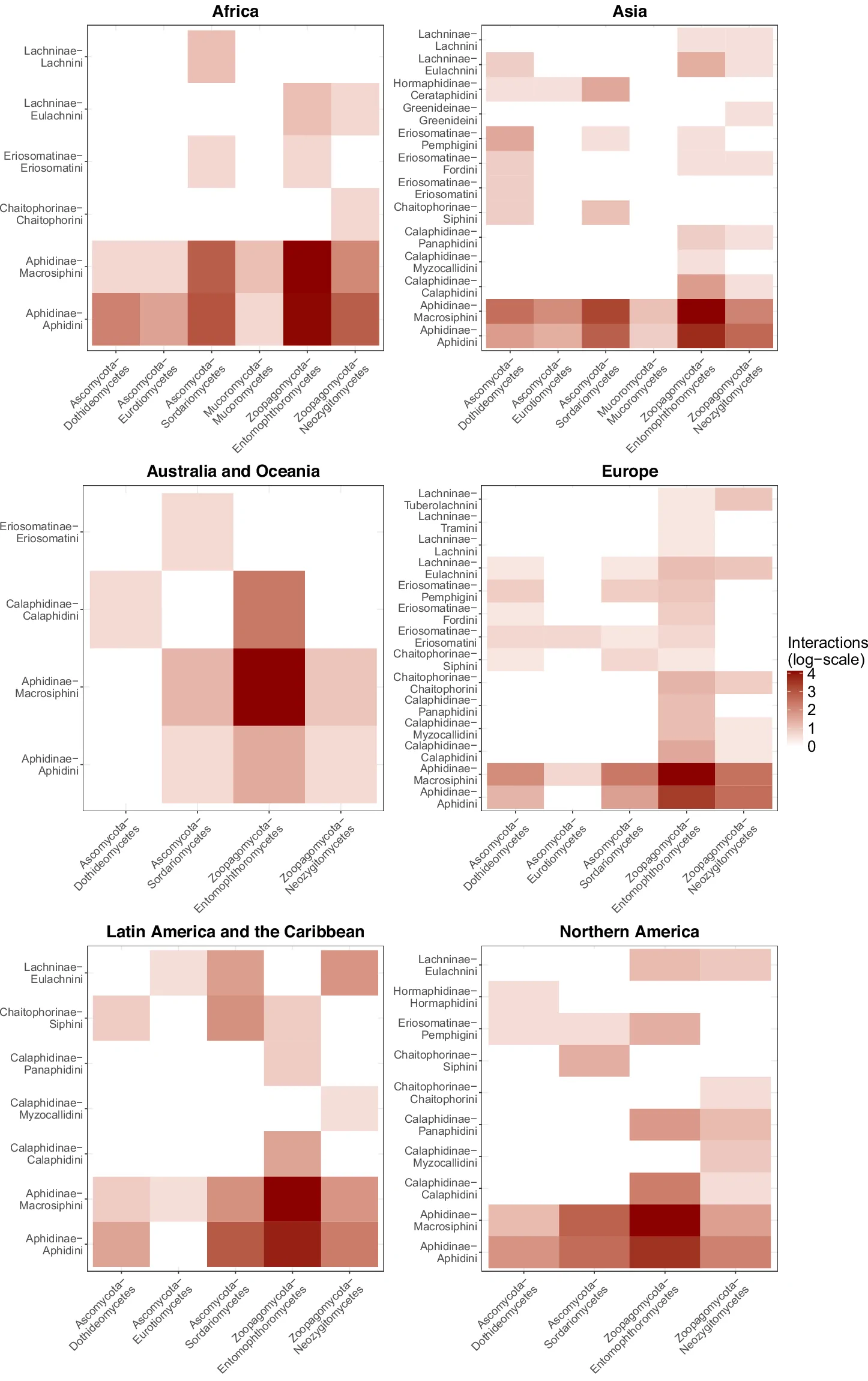
Frequency of entomopathogenic interactions, measured as the number of unique species-to-species links, between aphid tribes and fungal classes.

With this diverse taxonomic coverage, the AphidFunga database is particularly valuable for including information published in non-English languages and providing updated taxonomic classification, even when records originate from historical publications with past nomenclature. The aphid-fungus associations reported in AphidFunga are comprehensive yet geographically biased, with an overrepresentation of records from developed countries (Fig. 5). At the species level, the highest diversity of pathogenic records was reported from Europe, with 736 aphid–fungal interactions, followed by Asia (479), Latin America and the Caribbean (299), North America (262), Africa (164), and Australia and Oceania (94). These patterns likely reflect both differences in the susceptibility of aphid tribes to fungal infection and the uneven distribution of research efforts across continents.

## Materials and Methods

### Data compilation

A preliminary list of published interactions between aphids and fungi was queried from the Global Biotic Interaction (GloBI) database, with the help of the rglobi R package^33^. Next, each logged aphid-fungus interaction was traced back to its respective literature source from which additional data (e.g., location) were extracted.

A series of literature searches, with search strings containing aphid (aphids) AND fungus (fungi) were then executed in several academic databases, such as Scopus and Web of Science, and search engines like Google Scholar, Bing, and Baidu. The grey literature was covered by complementing the above searches with queries in eight non-English languages i.e., Arabic, Chinese, French, German, Hungarian, Polish, Russian, and Spanish. For each database, search engine and language, all search results were systematically collected and screened for relevance. Over 800 scientific papers and books published between 1856 and 2025 were systematically screened. For each publication, data were then manually extracted and stored in temporary data sheets. Scientific names of the interaction partners, the country where the interaction was recorded, the type of interaction as originally reported, the updated interaction type as aligned with current knowledge, and the source publication were recorded as per^28^. Only naturally occurring interactions between aphids and fungi were recorded; *in vitro* infections were excluded.

### Data processing

#### Data updates and formatting

**Fig. 6:**
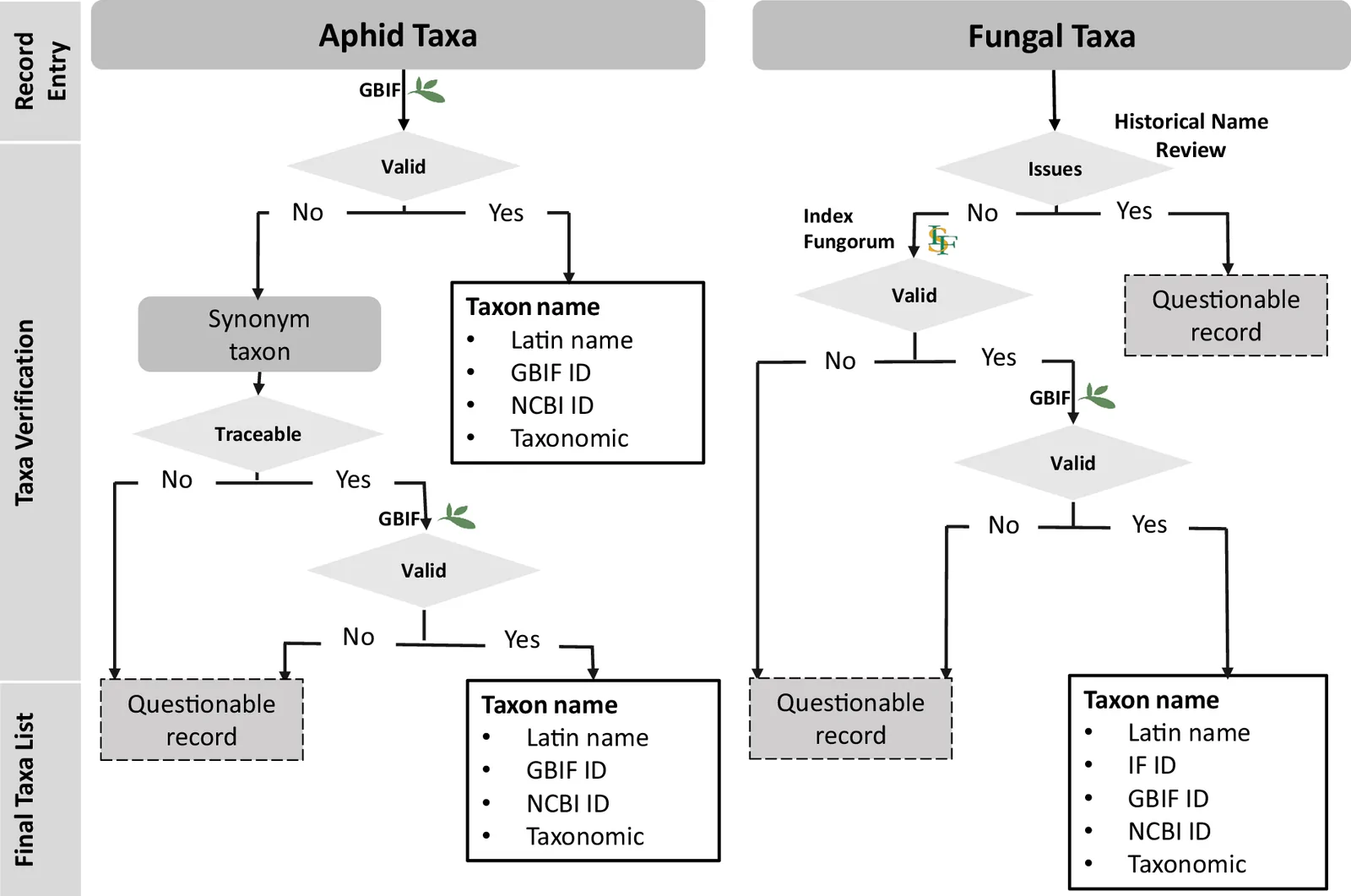
Flow chart describing the process used to validate records of aphids and associated fungal taxa.

The originally recorded taxon names were updated to currently valid names, in most cases through a custom R code exploiting the name_backbone() and get_ids() functions of the rgbif^46^ and taxize^47^ packages, respectively. The GBIF Taxonomy Backbone^48^ was used to verify the validity of the recorded aphid names. Fungal species names were checked against Index Fungorum^49^ and updated to their currently valid names, when necessary. Finally, the updated fungal names were matched to the GBIF database to obtain their corresponding GBIF IDs. When the automated taxonomic search failed, names were manually curated and a GBIF or Index Fungorum identifier was assigned to each except for two aphid species, *Myzus velutini* and *Prociphilus medificus*, for which currently accepted names could not be found; these were consequently classified under “Unknown species” (Fig. 6). These two species were given manually assigned IDs, their higher taxonomy was obtained from the genera, and were retained in the database and included in summaries and figures in this article.

Source publications were imported to Zotero^50^ during the data collection process. These records were then exported from Zotero’s SQLite database and matched with the data records through their unique identifiers. For each aphid-fungus interaction, unique combinations with the country of record were identified, and the first publication documenting each interaction–country pair was recorded. Only the first publication record was retained in the final database. Data cleaning, queries, and writing to MySQL tables were done in an R environment^51^(R Core Team 2012), with the help of dplyr^52^, reshape^53^, and RMySQL^54^ packages.

#### Organizing data and decisions

The AphidFunga dataset highlights the variation in the pathogenicity spectrum of fungi towards aphids. We sorted the aphid-fungal associations into four categories (Obligate aphid pathogens, Facultative aphid pathogens, Non-pathogens, and Undefined). “Obligate aphid pathogens” are strictly dependent on their aphid host for nutrients and survival and for fulfilling their life cycle. “Facultative aphid pathogens” include three fungal groups with different traits: (a) insect pathogens, where each species has a broader host range, not confined to aphids, (b) facultative insect pathogenic fungi which exhibit both saprobic and pathogenic lifestyles, and (c) opportunistic species with a pathogenic potential, i.e., causing disease only under specific conditions or when overabundant^13^. “Non-pathogens” refer to fungal species that occur and survive in different substrates but do not cause disease in insects. Saprobic fungi, plant pathogenic fungi, and endosymbionts belong to the latter category. Some associations could not be precisely categorised due to a lack of information on the fungal lifestyle; these were categorised as “Undefined” associations. These undefined associations can either be retained as a distinct category within the analytical pipeline or excluded from the dataset when the focus is on ecological function.

The taxonomy of aphid-pathogenic entomophthoralean fungi used to be unsettled, leading to confusion in species nomenclature and synonymy^17^. Although fungal features such as conidial shape, the formation of secondary conidia (capilliconidia), the presence and structure of cystidia, conidiophores, and rhizoids have long been accepted as key taxonomic criteria^18,55^, many historical reports lack detailed descriptions of the fungi collected. This has led to questionable and confusing records, particularly in genera with similar morphological features; for example, the similar shape of primary conidia in *Batkoa*, *Entomophaga*, and *Conidiobolus*. At the 15th Annual Meeting of the Society for Invertebrate Pathology (1982, University of Sussex, UK), the taxonomy of entomophthoralean fungi was discussed in depth due to several publications dealing with the subject^56–58^. It is therefore a natural choice for us to adopt 1980 as the dividing line for ‘modern descriptions’ of entomophthoralean taxa, from when authors have increasingly used revised genus names and elaborated species descriptions.

To overcome taxonomical limitations, AphidFunga includes (1) records published pre-1980, in which (a) species were attributed to debatable genera such as *Empusa*, *Entomophthora* sensu lato, and *Zoophthora* sensu lato, and (b) species synonyms which have been used interchangeably, for example *Entomophthora aphidis* and *Pandora neoaphidis* and (2) untraceable aphid and/or fungal taxa or those, which are considered invalid according to Index Fungorum and/or GBIF. Records dealing with these specific cases were flagged and notes on the uncertainty of identification were added. These records were retained in the database and included in all database-wide statistics, including total record counts, but excluded from specific analytical summaries and visualizations presented in this study.

Similarly to other fungal taxonomy databases, Index Fungorum^49^, although an internationally recognized global fungal nomenclatural database, may not always reflect the most current taxonomic status. Nevertheless, it remains a reliable and widely used resource. In line with modern nomenclature, we adopted the phylum Zoopagomycota instead of Entomophthoromycota^59^. At lower taxonomic ranks (e.g., class, order, family), classifications may vary depending on the source consulted. Aphid-pathogenic fungi are placed in two major clades, Ascomycota, and Zoopagomycota^59–61^, encompassing two insect pathogenic orders: Entomophthorales and Neozygitales. These two orders are often named together as “entomophthoralean fungi”, a term we adopt here. Nonetheless, AphidFunga is designed to remain flexible and open to incorporating future taxonomic revisions.

As a general approach, if associations from one country were recorded at a level of a higher taxon (normally genus), they were only kept if no more precise records were available from any member of that taxon. For instance, the interaction between *Rhopalosiphum padi* and the genus *Conidiobolus* recorded from Egypt would not have been kept if a report of the interaction between *R. padi* and a known *Conidiobolus* species (e.g. *C. coronatus*) from Egypt was present. In this case, the association between *R. padi* and *C. coronatus* would provide more precise information than the genus-level report, which, thus, would be excluded.

On several occasions, no current country could be assigned to the location where a given aphid-fungus association was reported from: 1) either the specific location was not reported at all; 2) the location was situated within a country that no longer exist (e.g. The Soviet Union); or 3) the geographic region reported as the sampling location spanned several countries (e.g. Transcaucasia which includes parts of Georgia, Armenia, and Azerbaijan). In these cases, we first checked the original source for any information detailed enough to assign the record to a specific country. If this was not possible, we chose the most likely option (e.g., the largest country or one where the author had previously reported from). In either case, when the country of origin was questionable, the originally reported text was added to the notes, and the country field was flagged. While it was impossible to determine the original country in 78 cases (2.6%), they are retained in the database, with the country of origin marked as ‘NA’. In cases when we gathered the same aphid-fungus association from a defined and an unknown location, the record with the unknown location was omitted.

## Data Records

To increase the usability of AphidFunga database^62^, we provide it in three different formats: 1) one large table, containing all data and one table containing all the notes and comments, in comma-separated files; 2) four separate comma-separated tables, as in a relational database, and 3) an importable SQL file from which both the linked tables and the connector tables can be directly imported to a MySQL database.

**Fig. 7:**
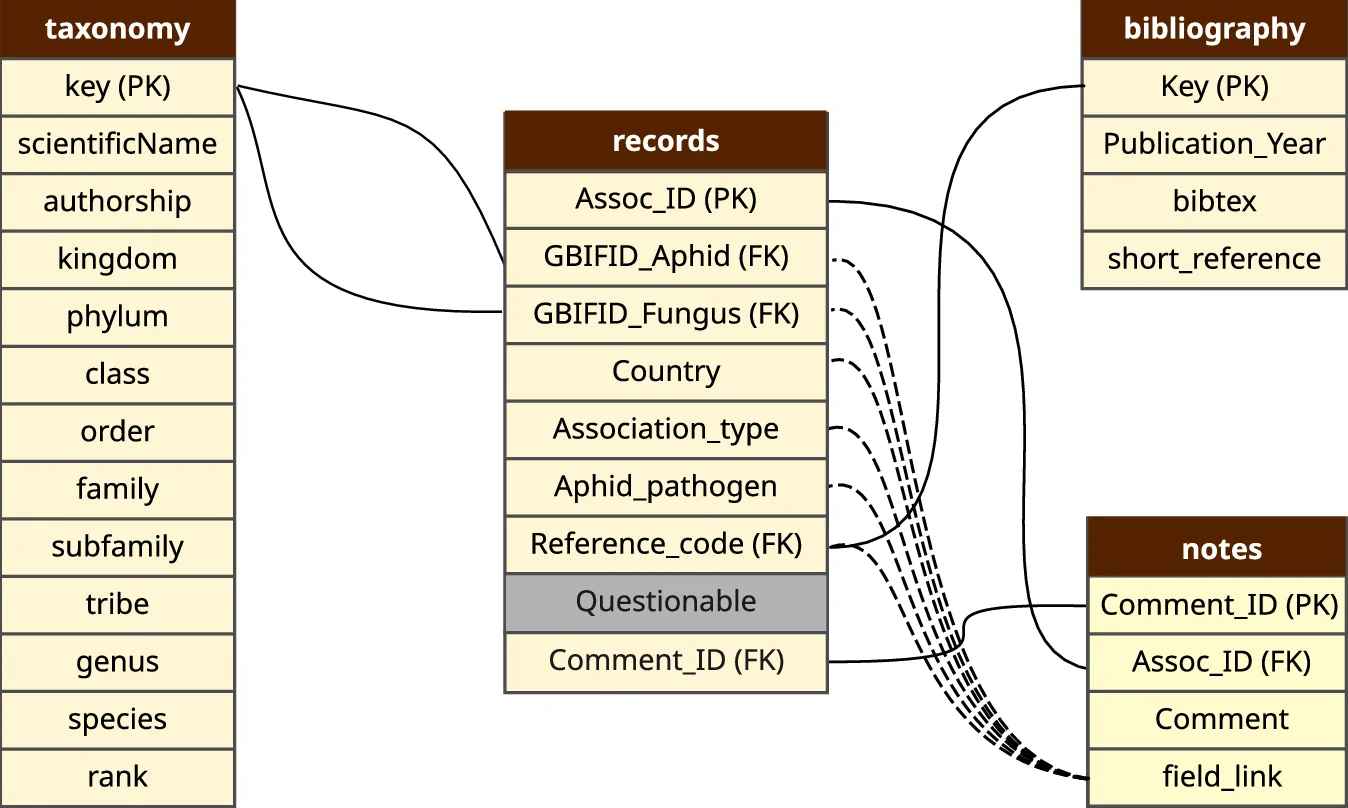
The structure of the MySQL database. Solid lines represent linked key fields between primary keys (PK) and foreign keys (FK). Dashed lines represent logical links between notes and the information piece they refer to. The greyed-out ‘Questionable’ field in the ‘records’ table is not included in the MySQL table but presented in the merged dataset.

The main table (‘*records*’) contains the recorded associations, as a unique line for each aphid identifier (GBIFID_Aphid), fungus identifier (GBIFID_Fungus), association type (Association_type), whether the association is considered pathogenic (Aphid_pathogen), the country where the association was recorded (Country), a unique identifier to the literature source (Reference_code), a field indicating which lines were flagged as questionable because of uncertainties in fungal taxonomy (see ‘*Organizing data and decisions*’), and a field listing all flagged identifiers pointing to individual comments explaining the issues (Comment_ID). In the MySQL database and the R data files, separate tables contain the taxonomy of the recorded species and the detailed bibliographic records, using unique rows for each species or literature source (Fig. 7). These tables are merged with the main table in the large comma-separated dataset using the same column names as in the separate tables (but rows can be repeated). The ‘*taxonomy*’ table contains the GBIF identifiers of the taxon (or, in the very few cases where we were unable to trace the taxon, a manually created unique identifier), the full scientific name, higher taxonomy (family, subfamily, tribe for aphids and kingdom, phylum, class, and order for fungi), the rank of the taxon (species or genus), and the authorship linked to the taxon. The ‘*bibliography*’ table contains a unique identifier (Key) for each reference, the year of publication, a short version of the reference (short_reference), as in-text citations, and the full reference in a BIBTEX format (Bibtex). On top of these, there is a separate table for all the flags and notes (‘*notes*’). This table is never merged with the data table, but it can be easily linked through the unique identifier of the data lines (Assoc_ID) and the name of the data field it is logically linked to.

## Technical Validation

The species names included in the database were both programmatically cross-checked against taxonomic databases and manually curated by experts. Data with uncertain taxonomies still remaining in the database were flagged to highlight the uncertainties. These uncertainties affected < 10% of the collected species names, and < 5% of the data. The authors validated association types, and questionable cases were determined by multi-expert consensus. Each data point was also subjected to manual checks and all the edits in the database were recorded and stored as metadata. The source literature was thoroughly checked for duplicates.

## Usage Notes

The AphidFunga database is equally accessible to scientists, stakeholders, and decision-makers dealing with ecosystems and pest management. This dataset lays the groundwork for further investigations of fungus-mediated ecosystem processes and functions and has great potential to be used in conservation agriculture and designing nature-based solutions for biological pest control. The information on the locations where the associations were reported facilitates making spatially informed biocontrol decisions based on current knowledge. From a biocontrol perspective, the dataset includes only naturally occurring aphid–fungal associations, and strain-level information, which is often relevant for applied use, is therefore not included in the database. However, additional details of potential relevance for agricultural applications may be found in the original literature sources accessible via the database references.

Owing to its structured relational design, the database is straightforward to update, query, adapt to alternative formats and merge with other databases. Additionally, each taxon has been verified and linked to unique identifiers of major external databases, enabling easy integration with other international resources. Bibliographic information is provided both in a human-readable APA citation style and as BibTeX entries, supporting seamless identification of the literature resources.

## Supplementary information

Supplementary information

## Data Availability

The three different formats of AphidFunga are available on figshare: https://doi.org/10.6084/m9.figshare.30813824.

## Code availability

All computer codes used to generate and clean the AphidFunga database are freely available at https://github.com/pozsgaig/AphidFunga.
