# Supplementary material for: A curated global dataset of naturally occurring aphid-fungal associations
Source: Sci Data. 2026 Jul 1;13:1583. doi: 10.1038/s41597-026-07691-5 (PMC13754900; doi:10.1038/s41597-026-07691-5)
Supplement: Supplementary file 1 [file 41597_2026_7691_MOESM1_ESM.pdf]

Supplementary reference list for the AphidFunga database

Abdel-Baky, N.F., 2000. *Cladosporium* spp. An entomopathogenic fungus for controlling whiteflies and aphids in egypt. Pakistan Journal of Biological Sciences 3, 1662–1667. https://doi.org/10.3923/pjbs.2000.1662.1667

Abdel-Mallek, A.Y., Abdel-Rahman, M.A.A., Omar, S.A., Hamam, G.H.A., 2004. A comparative abundance of entomopathogenic fungi of cereal aphids in assiut, egypt, in: Proceeding 2nd Saudi Science Conference. pp. 167–174.

Agriculture, U.S.D. of, Agricultural Research Service, B.I.P.M.R.U., 2016. ARS collection of entomopathogenic fungal cultures (ARSEF).

Ahmad, M., Rajab, L., 2018. First record of entomopathogenic fungus *neozygites fresenii* on *aphis fabae* in syria. Arab and Near East Plant Protetion Newsletter 75, 8.

Alaserhat, I., 2015. Erzincan ve gümüshane illerinde yetistirilen iliman iklim meyve türlerinde bulunan aphididae (hemiptera) türleri, yogunluklari, dogal düsmanlari ve sekonder konukçularinin belirlenmesi (PhD thesis). Atatürk Üniversitesi, Erzurum, Turkey.

Alemán, J., Ravelo, J., A. Martínez, M. de los, 2008. Fungus epizootic detected on *lipaphis erysimi* populations in two cuban provinces. Revista de Protección Vegetal 23, 196.

Alzugaray, R., Ribeiro, A., Silva, H., Stewart, S., Castiglioni, E., Bartaburu, S., Martínez, J.J. y, 2010. Prospeccin de agentes de mortalidad natural de áfidos en leguminosas forrajeras en uruguay. Agrociencia 14, 27–35.

Andrade, M.E., Briceño, J.A., Muños de Hoyos, P., Jiménez, J., 1989. Búsqueda y reconocimiento de los enemigos naturales y hospedantes alternos de las principales plagas. En flores bajo invernadero en la sabana de bogotá. Acta Biológica Colombiana 1, 45–57.

Anonymous, 1987. Enfermedades en insectos causadas por hongos. Corporación Colombiana de Investigación Agropecuaria.

Apablaza, J.H.U., Tiska, V.V., 1973. Poblaciones de áfidos (homoptera: Aphididae) en trigo de la zona central chilena. Revista Chilena de Entomología 7, 173–180.

Araújo Filho, J.M. de, 2008. Seleção de fungos entomopatogênicos associados ao óleo de nim para controle do pulgão lipaphis erysimi (kalt.) (hemiptera : Aphididae) em couve (PhD thesis). Universidade Federal Rural de Pernambuco, Brasil.

Aruta, C., Carrillo, R., 1989. Identificación de hongos del orden entomophthorales en chile III. Agro Sur 17, 10–18.

Aruta, C., Carrillo, R., González, S., 1974. Determinacion para chile de hongos entomopatogenos del genero *entomophthora*. i. Agro sur 2, 62–70.

Asan, A., 2011. Checklist of *fusarium* species reported from turkey. Mycotaxon 116, 479.

Asante, S.K., 1997. Natural enemies of the woolly apple aphid, eriosoma lanigerum (hausmann) (hemiptera: Aphididae): A review of the world literature. Plant Protection Quarterly 12, 166–172.

Aswini, G.V., Manjuatha, M., Naik, M.I., 2007. Occurrence of fungal pathogens on sugarcane woolly aphid *ceratovacuna lanigera* zehntner in bhadra command area. Karnataka Journal Agricultural Science 20, 764–766.

Austarå, Ø., Carter, C., Eilenberg, J., Halldórsson, G., Harding, S., 1997. Natural enemies of the green spruce aphid in spruce plantations in maritime north-west europe. Búvísindi 11, 113–124.

Baiswar, P., Firake, D.M., Ngachan, S., Chandra, S., Thakur, N.S.A., 2016. *Lecanicillium longisporum* on cabbage aphid (*brevicoryne brassicae*): A new record from northeast india. National Academy Science Letters 39, 311–315. https://doi.org/10.1007/s40009-016-0452-2

Balazy, S., 2003. On some little known epizootics in noxious and beneficial arthropod populations caused by entomophthoralean fungi. International Organisation for Biological and Integrated Control/West Palaearctic Regional Section Bulletin 26, 63–68.

Balazy, S., 1993. Entomophthorales, in: Flora of Poland. Fungi (Mycota). Polish Academy of Sciennce, pp. 1–356.

Balazy, S., 1985. Notes on *hirsutella aphidis*. Transactions of the British Mycological Society 85, 752–756.

Balazy, S., Mietkowski, R., Majchrowicz, I., 1990. Mikozy mszyc - ich znaczenie i perspektywy wykorzystania w ochronie roslin. Zeszyty Problemowe Postepów Nauk Rolniczych 392, 35–56.

Barta, M., 2009b. Entomophthoralean fungi associated with aphids in woody plants in the arboretum mlynany SAS. Folia Oecologica 36, 1–7.

Barta, M., 2009a. Entomopatogénne huby z radu entomophthorales v populáciách vosiek na drevinách v mestskom prostredí. Slovenská spolocnost pre vedy pol'nohospodárske, lesnícke, potravinárske a veterinárske pri SAV Bratislava, Pobocka Nitra, Nitra.

Barta, M., 2004. Fungi of the order entomophthorales infecting aphids in slovakia (PhD thesis). Slovak University of Agriculture in Nitra, Nitra.

Barta, M., Cagán, L., 2006. Aphid-pathogenic entomophthorales (their taxonomy, biology and ecology). Biologia 61, S543–S616. https://doi.org/10.2478/s11756-007-0100-x

Barta, M., Cagán, L., 2004. A potential role of *rhopalosiphum padi* (linneus) colonies on winter host, *padus avium* linneus, as an inoculum source of fungal diseases of cereal aphids in agricultural landscape. Acta Fytotechnica et Zootechnica 7, 22–25.

Barta, M., Cagán, L., 2003a. *Pandora uroleuconii* sp. Nov. (Zygomycetes: Entomophthoraceae), a new pathogen of aphids. Mycotaxon 88, 79–86.

Barta, M., Cagán, L., 2003b. Entomophthoralean fungi associated with the common nettle aphid (*microlophium carnosum* buckton) and the potential role of nettle patches as reservoirs for the pathogens in landscape. Anzeiger für Schädlingskunde 76, 6–13. https://doi.org/10.1046/j.1439-0280.2003.03004.x

Barta, M., Cagán, L., 2002. Prevalence of natural fungal mortality of black bean aphid, *aphis fabae* scopoli on primary host and two secondary hosts. Acta Fytotechnica et Zootechnica 5, 57–64.

Barta, M., Keller, S., Cate, P., Wegensteiner, R., 2005. Observations on the occurrence of entomophthorales in austria. International Organisation for Biological and Integrated Control/West Palaearctic Regional Section Bulletin 28, 49–52.

Barta, M., Stalmachová-Eliasová, M., Alexandra, Hozzank, Ludovic, Cagán, Rudolf, Wegensteiner, 2003. Results of preliminary investigations on the occurrence of entomophthorales on aphids in austria. International Organisation for Biological and Integrated Control/West Palaearctic Regional Section Bulletin 26, 81–83.

Batista, L., Porras, D.N., Gutiérrez, A., Peña, I., Rodríguez, J., Del Amo, O.F., Pérez, R., Lee, R.F., Niblett, C.L., Morera, J.L., 1996. Tristeza and *toxoptera citricida* in cuba: Incidence and control strategies. International Organization of Citrus Virologists Conference Proceedings (1957-2010) 13, 104–111.

Batko, A., 1964. Notes on entomophthoraceous fungi in poland, in: Entomophaga. Paris, France, pp. 129–131.

Batra, H.N., Kumar, K., 1962. Occurence of green peach aphid, *myzus persicae* sulzer as a serious pest of pansy flower and its control. Indian journal of entomology 23, 149–151.

Ben Fekih, I., Boukhris-Bouhachem, S., Bechir Allagui, M., Bruun Jensen, A., Eilenberg, J., 2015. First survey on ecological host range of aphid pathogenic fungi (phylum entomophthoromycota) in tunisia. Annales de la Société entomologique de France 51, 140–144. https://doi.org/10.1080/00379271.2015.1059997

Ben Fekih, I., Boukhris-Bouhachem, S., Eilenberg, J., Allagui, M.B., Jensen, A.B., 2013. The occurrence of two species of entomophthorales (entomophthoromycota), pathogens of sitobion avenae and myzus persicae (hemiptera: Aphididae), in tunisia. BioMed Research International 2013, 838145. https://doi.org/10.1155/2013/838145

Bensch, K., Braun, U., Groenewald, J.Z., Crous, P.W., 2012. The genus *cladosporium*. Studies in Mycology 72, 1–401. https://doi.org/10.3114/sim0003

Ben-Ze'ev, Israel, Kenneth, R.G., 1981. *Zoophthora orientalis* sp. Nov., a fungal pathogen of *aphis citricola* (homoptera: Aphididae), and two new combinations of other species of entomophthoraceae. Phytoparasitica 9, 33–42. https://doi.org/10.1007/BF03158327

Ben-Ze'ev, I., Kenneth, R.G., 1981. *Zoophthora radicans* and *zoophthora petchi* sp. Nov. [Zygomycetes: Entomophthorales], two species of the "*sphaerosperma* group" attacking leaf-hoppers and frog-hoppers [hom.]. Entomophaga 26, 131–142. https://doi.org/10.1007/BF02375027

Ben-Ze'ev, I., Kenneth, R.G., 1979. *Zoophthora erinacea* sp. N. (Zygomycetes: Entomophthoraceae), a fungal parasite of aphids. Mycotaxon 10, 219–232.

Ben-Ze'ev, I.S., 1993. Check-list of fungi pathogenic to insects and mites in israel, updated through 1992. Phytoparasitica 21, 213–237. https://doi.org/10.1007/BF02980944

Ben-Ze'ev, I.S., Jaques, R.P., 1990. Entomopathogenic fungi in insects in alfalfa fields in southwestern ontario. Proceedings of the Entomological Society of Ontario 121, 71–78.

Ben-Ze'ev, I.S., Uziel, A., 1979. *Monellia costalis* (fitch), a new host for *zoophthora radicans* (brefeld) batko and *entomophthora planchoniana* cornu (zygomycetes: Entomophthoraceae) in israel. Phytoparasitica 7, 159–167. https://doi.org/10.1007/BF02981122

Berlanga-Padilla, A.M., Ayala-Zermeño, M.Á., Gallou, A., Serna-Domínguez, M.G., Montesinos-Matías, R., Arredondo-Bernal, H.C., 2016. Identificación de *lecanicillium longisporum* asociado a *melanaphis sacchari* (hemiptera: Aphididae), en sorgo. Revista Mexicana de Micología 44, 51–54.

Bitton, S., Kenneth, R.G., Ben-Ze'ev, I.S., 1979. Zygospore overwintering and sporulative germination in *triplosporium fresenii* (entomophthoraceae) attacking *aphis spiraecola* on citrus in israel. Journal of Invertebrate Pathology 34, 295–302. https://doi.org/10.1016/0022-2011(79)90076-4

Bode, E., 1980. Untersuchungen zum auftreten der haferblattlaus *rhopalosiphum padi* (l.) (homoptera: Aphididae) an ihrem winterwirt *prunus padus* l. Zeitschrift für Angewandte Entomologie 89, 363–377. https://doi.org/10.1111/j.1439-0418.1980.tb03477.x

Bode, E., 1977. Cereal aphids and their antagonists in 1977, in: Reunion o.i.l.b. Of the Group "Integrated Control in Cereals", Subgroup "Ecology of Cereal Aphids". Zürich, Switzerland.

Börner, C.J.B., Heinze, K., 1957. Handbuch der pflanzenkrankheiten, in: Sorauer, P. (Ed.), Handbuch Der Pflanzenkrankheiten. Paul Parey, Berlin, Hamburg, pp. 187–199.

Braxton, S.M., Onstad, D.W., Dockter, D.E., Giordano, R., Larsson, R., Humber, R.A., 2003. Description and analysis of two internet-based databases of insect pathogens: EDWIP and VIDIL. Journal of Invertebrate Pathology 83, 185–195. https://doi.org/10.1016/S0022-2011(03)00089-2

Buchner, P., 1912. Studien an intracelularen symbionten - 1. Die intracellularen symbionten der hemipteren. Archiv für Protistenkunde 26, 1–116.

Buckton, G.B., 1879. Monograph of the british aphides. London,Printed for the Ray Society.

Burger, O.F., Swain, A.F., 1918. Observations on a fungus enemy of the walnut *aphis* in southern california. Journal of Economic Entomology 11, 278–289. https://doi.org/10.1093/jee/11.3.278

Bustillo Pardey, A.E., 2011. Parasitoides, predadores y entomopatógenos que afectan las plagas de la caña de azúcar en colombia. Centro de Investigación de la Caña de Azúcar de Colombia 719.

Bustillo Pardey, A.E., 2001. Hongos en insectos y posibilidades de uso en el control biológico de plagas en colombia, in: Uso de Entomopatógenos En Cololombia. Instituto de Ciencias Naturales, UN Colombia, Colombia, pp. 30–53.

Byford, W.J., Reeve, G.J., 1969. Entomophthora species attacking aphids in england, 1962-66. Transactions of the British Mycological Society 52, 342–346. https://doi.org/10.1016/S0007-1536(69)80050-1

Cagán, L., Barta, M., 2001. Seasonal dynamics and entomophthoralean infection of the pea aphid, *acyrthosiphon pisum* harris. Plant Protection Science 37, 17–24. https://doi.org/10.17221/8363-PPS

Cameron, P.J., Milner, R.J., 1981. Incidence of *entomophthora* spp. In sympatric populations of *acyrthosiphon kondoi* and *a. Pisum.* New Zealand Journal of Zoology 8, 441–446. https://doi.org/10.1080/03014223.1981.10430625

Campbell, R.E., 1926. The pea aphid in california. Journal of Agricultural Research 32, 861–881.

Carl, K.P., Mohyuddin, I., 1989. Agriculture canada annual project report 1989 - explorations on russion wheat aphid in 1989 and collection of natural enemies. Commonwealth Agricultural Bureaux, International Institute of Biological Control, European Station, Delémont, Switzerland. Reoprt 1989 20–22.

Carrillo Li., R., Mellado Z.., M., Pino B., A., 1974. Los áfidos *sitobion avenae* (fab.) y *metopolophium dirhodum* (walk.), su influencia en el rendimiento, ubicacion en la planta y sus enemigos naturales. Agro Sur 2, 71–85. https://doi.org/10.4206/agrosur.1974.v2n2-07

Charles, V.K., 1941. A reliminary check list of the entomogenous fungi of north america. The Insect Pest Survey Bulletin - Bureau of Entomology and Plant Quarantine United States Department of Agriculture and the State Entomological Agencies Cooperating 21, 707–785.

Chen, B., Li, Z.Y., Feng, M.G., 2008. Occurrence of entomopathogenic fungi in migratory alate aphids in yunnan province of china. BioControl 53, 317–326. https://doi.org/10.1007/s10526-006-9063-z

Chen, C., Feng, M., 2002. Evidence for transmission of aphid-pathogenic fungi by migratory flight of *myzus persicae* alates. Chinese Science Bulletin () 47, 1987–1989. https://doi.org/10.1360/02tb9431

Cheng, S.Q., Long, H.R., 1987. Study on conidiobolus osmodes as a pathogen of the soybean aphid: Isolation, cultivation, and identification. Acta Mycologica Sinica 6, 129–132.

Cinovskij, J.P., Egina, K.J., 1972. Ispolzowanie entomoftoralnych gribov v borbe s tljami, in: Cinovskij, J.P., Egina, K.J., Simonova, E.Z. (Eds.), Patologija Nasekomyh i Klescej. [s.n.], Riga, pp. 72–94.

Comerio, R.M., Andorno, A.V., Botto, E.N., 2008. Aislamiento de *conidiobolus coronatus* a partir de una plaga de áfidos del cebollín (*allium schoenoprasum* l.). Revista Iberoamericana de Micología 25, 193–195. https://doi.org/10.1016/S1130-1406(08)70046-9

Cornu, M., 1873. Note sur une nouvelle espece d'*entmnophthora* (*e. Planchoniana*). Bulletin de la Société Botanique de France 20, 189–191.

Cortez-Madrigal, H., Ortiz-García, C.F., Alatorre-Rosas, R., Bravo-Mojica, H., Mora-Aguilera, G., Aceves-Navarro, L.A., 2003. Caracterización cultural de cepas de *lecanicillium* (=*verticillium*) *lecanii* (zimm.) zare y gams y su patogenicidad sobre *toxoptera aurantii* boyer. Revista Mexicana de Fitopatología 21, 161–167.

Cotton, A.D., 1919. Entomogenous fungi new to britain. Transactions of the British Mycological Society 6, 200–203. https://doi.org/10.1016/S0007-1536(17)80029-2

Cudare, Z., 1998. Biodiversity of entomopathogenic fungi in latvia and their potential in plant protection. International Organisation for Biological and Integrated Control/West Palaearctic Regional Section Bulletin 21, 85–88.

Dara, S.K., Semtner, P.J., 2001. Incidence of *pandora neoaphidis* (remaudière and hennebert) humber (zygomycetes: Entomophthorales) in the *myzus persicae* (sulzer) complex (homoptera: Aphididae) on three species of brassica in the fall and winter. Journal of Entomological Science 36, 152–161. https://doi.org/10.18474/0749-8004-36.2.152

Day, K., 1984. The growth and decline of a population of the spruce aphid *elatobium abietinum* during a three year study, and the changing pattern of fecundity, recruitment and alary polymorphism in a northern ireland forest. Oecologia 64, 118–124. https://doi.org/10.1007/BF00377553

Dean, G.J.W., Wilding, N., 1973. Infection of cereal aphids by the fungus *entomophthora*. Annals of Applied Biology 74, 133–138. https://doi.org/10.1111/j.1744-7348.1973.tb07732.x

Dean, G.J.W., Wilding, N., 1971. *Entomophthora* infecting the cereal aphids *metapolophium dirhodum* and *sitobion avenae*. Journal of Invertebrate Pathology 18, 169–176. https://doi.org/10.1016/0022-2011(71)90144-3

Dedryver, C.-A., 1978. Rôle de l'humidité relative sur le développement du champignon parasite, *entomophthora aphidis* hoffmann, dans les populations de l'aphide, *sitobion avenae* f., en 1976, dans l'ouest de la france. Comptes Rendus de l'Académie des Sciences 286, 1723–1726.

Dedryver, F., Rabasse, J.M., 1977. Repartition spatiale et developpement des populations du puceron noir de la fève (aphis fabae scop.) en zone bocagère et en zone arasée. Incidence des entomophthora, in: C. R. Table Ronde c. N. R. S. « Ecosystème Bocagère », Rennes 1976. pp. 367–375.

Díaz Densani, B.M., Legarrea, S., Marcos-García, M. ngeles, Fereres, A., 2010. The spatio-temporal relationships among aphids, the entomophthoran fungus, *pandora neoaphidis*, and aphidophagous hoverflies in outdoor lettuce. Biological Control 53, 304–311. https://doi.org/10.1016/j.biocontrol.2009.12.002

Díaz Desani, B.M., López Lastra, C.C., Oggerin, M., Fereres, A., Rubio, V., 2008. Identificación de hongos entomopatógenos asociados a pulgones en cultivos hortícolas en la zona centro de la península ibérica. Boletin de Sanidad Vegetal Plagas 34, 287–296.

Dinu, M.M., Fatu, A.-C., Andrei, A.-M., 2015. Natural outbreak of an entomophthoralean fungus, *pandora neoaphidis* on *macrosiphum* sp. Romanian Journal for Plant Protection 8, 78–81.

Doidge, E.M., 1950. The south african fungi and lichens. Bothalia 5, 1–1094. https://doi.org/10.4102/abc.v5i1.1872;10.4102/abc.v5i1.1873

Draganova, S., Simova, S., 2001. Entomopathogenic fungi on *aphis pomi* de geer (homoptera: Aphididae) and *tetranychus urticae* k. (Acarina: tetranychoidea). Bulgarian Journal of Agricultural Science 7, 595–599.

Dubiel, G., 2016. Wstepna dane na temat wystepowania owadomorkowców (enomophthorales) w beskidzie slaskim. Przeglad Przyrodniczy 27, 56–69.

Dunn, J.A., Kempton, D.P.H., 1971. Seasonal changes in aphid populations on brussels sprouts. Annals of Applied Biology 68, 233–244. https://doi.org/10.1111/j.1744-7348.1971.tb04642.x

Edwards, T.L., 1981. An entomophthora species on *cinara pilicornis* (hartig) (hemiptera: aphididae). The Irish Naturalists' Journal 20, 204–206.

Ekbom, B.S., Pickering, J., 1990. Pathogenic fungal dynamics in a fall population of the blackmargined aphid (*monellia caryella*). Entomologia Experimentalis et Applicata 57, 29–37. https://doi.org/10.1111/j.1570-7458.1990.tb01413.x

Eken, C., Demirci, E., 1997. Funguslarin biyolojik mücadelede kullanimi. Atatürk Üniversitesi Ziraat Fakültesi Dergisi 28, 138–152.

Ekesi, S., Akpa, A.D., Onu, I., Ogunlana, M.O., 2000. Entomopathogenicity of *beauveria bassiana* and *metarhizium anisopliae* to the cowpea aphid, *aphis craccivora* koch (homoptera: aphididae). Archives Of Phytopathology And Plant Protection 33, 171–180. https://doi.org/10.1080/03235400009383341

El-Heneidy, A.H., Adly, D., 2012. Cereal aphids and their biological control agents in egypt. Review article. Egyptian Journal of Biological Pest Control 22, 227–244.

Eliasová, M., Papierok, B., Cagán, L., 2004. Mortality of *metopolophium dirhodum* (homoptera: Aphididae) caused by strains of *erynia neoaphidis* (entomophthorales: Entomophthoraceae) from slovakia and greece. Plant Protection Science 40, 54–62. https://doi.org/10.17221/2724-PPS

Elkassabany, N.M., Steinkraus, D.C., McLeod, P.J., Correll, J.C., Morelock, T.E., 1992. *Pandora neoaphidis* (entomophthorales: Entomophthoraceae): A potential biological control agent against *myzus persicae* (homoptera: Aphididae) on spinach. Journal of the Kansas Entomological Society 65, 196–199.

El-Maraghy, S.M.A., Mohamed, A.H.M., Abdel-Rahman, M.A.A., Awad, A.M.A., Omar, Y.M., 2015. Incidence of entomopathogenic fungi of the oat bird-cherry aphid, *rhopalosiphum padi* l. (Homoptera: Aphididae) infesting wheat plants in assiut, egypt. Journal of Basic & Applied Mycology 6, 43–47.

El-Shami, I.A., El-Sheikh, M.F.M., 2018. Survey of entomopathogenic fungi and parasitoids on two aphid species on wheat crop in gharbia governorate, egypt. Journal of Plant Protection and Pathology 9, 153–158. https://doi.org/10.21608/jppp.2018.41282

Embaby, E.S.M., Lotfy, D.E.S., 2015. Ecological studies on cabbage pest. International Journal of Agricultural Technology 11, 1145–1160.

Essig, E.O., 1926. Insects of western north america. The Macmillan Company, New York.

Fadayivata, S., Moravvej, G., Karimi, J., 2014. Pathogenicity of the fungus *lecanicillium longisporum* against *sipha maydis* and *metopolophium dirhodum* in laboratory conditions. Journal of Plant Protection Research 54, 67–73. https://doi.org/10.2478/jppr-2014-0010

Fan, M.-Z., Guo, C., Li, Z.-Z., 1991. [New species and a new record of the genus *erynia* in china]. Acta Mycologica Sinica 10, 95–100. https://doi.org/10.13346/j.mycosystema.1991.02.003

Farlow, W.G., Seymour, A.B., 1888. A provisional host-index of the fungi of the united states. Cambridge.

Fawcett, H.S., 1910. An important entomogenous fungus. Mycologia 2, 164–168. https://doi.org/10.2307/3753100

Feng, M.-G., Chen, C., Chen, B., 2004. Wide dispersal of aphid-pathogenic entomophthorales among aphids relies upon migratory alates. Environmental Microbiology 6, 510–6. https://doi.org/10.1111/j.1462-2920.2004.00594.x

Feng, M.-G., Johnson, J.B., Halbert, S.E., 1991a. Natural control of cereal aphids (homoptera: Aphididae) by entomopathogenic fungi (zygomycetes: Entomophthorales) and parasitoids (hymenoptera: Braconidae and encyrtidae) on irrigated spring wheat in southwestern idaho. Environmental Entomology 20, 1699–1710. https://doi.org/10.1093/ee/20.6.1699

Feng, M.-G., Johnson, J.B., Kish, L.P., 1990. Survey of entomopathogenic fungi naturally infecting cereal aphids (homoptera: Aphididae) of irrigated grain crops in southwestern idaho. Environmental Entomology 19, 1534–1542. https://doi.org/10.1093/ee/19.5.1534

Feng, M.-G., Nowierski, R.M., Scharen, A.L., Sands, D.C., 1991b. Entomopathogenic fungi (zygomycotina: Entomophthorales) infecting cereal aphids (homoptera: Aphididae) in montana. The Pan-Pacific Entomologist 67, 55–64.

Ferron, P., Brader, L., 1973. Souches de microorganismes entomopathogènes. International Organisation for Biological and Integrated Control/West Palaearctic Regional Section Bulletin 3, 1–30.

Folsom, J.W., 1909. The insect pests of clover and alfalfa. University of Illinois Agricultural Experiment Station, Urbana, Illinois, USA.

Freimoser, F.M., Jensen, A.B., Tuor, U., Aebi, M., Eilenberg, J., 2001. Isolation and in vitro cultivation of the aphid pathogenic fungus *entomophthora planchoniana*. Canadian journal of Microbiology 47, 1082–7.

https://doi.org/10.1139/w01-115
Fresenius, J.B.G.W., 1858. Über die pilzgattung *entomophthora*. Abhandlungen, herausgegeben von der Senckenbergischen Naturforschenden Gesellschaft 2, 201–210.
Gabriel, B.P., 1968. Entomogenous microorganisms in the philippines: New and past records. The Philippine Entomologist 1, 97–130.
Gao, R.X., Lin, G.X., Guan, X., 1986. A list of fujian pathogenic microbes on pests. Journal of Fujian Agricultural College 15, 300–310.
Ghosh, A.K., Ghosh, L.K., 2006. The fauna of india and the adjacent countries. Homoptera: aphidoidea. Zoological Survey of India, Kolkata, India.
Ghosh, L.K., 2008. Handbook on hemipteran pests in india. Zoological Survey of India, Kolkata.
Giard, A., 1889. Sur quelques types remarquables de champignons entomophytes. Bulletin Scientifique de la France et de la Belgique 20, 197–224.
Gilbert, E.M., Kuntz, W.A., 1926. Some diseases of *aphis spiraecola* patch. The Quarterly bulletin of the State Plant Board of Florida 10, 1–6.
Gindin, G., Ben-Ze'ev, I., 1994. Natural occurrence of and inoculation experiments with *conidiobolus coronatus* and *conidiobolus* sp. In glasshouse populations of *bemisia tabaci*. Phytoparasitica; Israel journal of plant protection sciences 22, 197–208.
Glare, T.R., Milner, R.J., Chilvers, G.A., Mahon, R.J., Brown, W.V., 1987. Taxonomic implications of intraspecific variation amongst isolates of the aphid-pathogenic fungi *zoophthora radicans* brefeld and *z. Phalloides* batko (zygomycetes, entomophthoraceae). Australian Journal of Botany 35, 49–67. https://doi.org/10.1071/BT9870049
Glare, T.R., O'Callaghan, M., Wigley, P.J., 1993. Checklist of naturally occurring entomopathogenic microbes and nematodes in new zealand. New Zealand Journal of Zoology 20, 95–120. https://doi.org/10.1080/03014223.1993.10422867
Godfrey, K., Steinkraus, D., McGuire, M., 2001. Fungal pathogens of the cotton and green peach aphids in the san joaquin valley. Southwestern Entomologist 26, 297–302.
Goettel, M.S., Erlandson, M.A., 1991. Fungal pathogens of cereal aphids in saskatchewan and alberta. Agric. Can. Biocontrol News 4, 45.
Goettel, M.S., Sigurdson, B.D., Yu, D.S., Butts, R.A., 1993. Native pathogens affect russian wheat aphid populations in southern alberta. Biocontrol News 6, 59–61.
Goettel, M.S., Yu, D.S., Duke, G.M., Erlandson, M.A., 1990. Potential of using biological control for the russian wheat aphid, *diuraphis noxia* in canada. Agric. Can. Biocontrol News 3, 32–39.
Gómez de Picho, H., 1992. Hongos entomopatógenos (zygomycetes: Entomophthoraceae) registrados parasitando pulgones (homoptera: aphididae). Revista Peruana de Entolomogía 35, 45–48.
Gres, Y., Koval, E.Z., 1982. A new species entomorphthora terrestris sp. Nova. Affecting the sugar-beet root aphid. Mikrobiologicheskii zhurnal 44, 64–69.
Grobler, J.H., MacLeod, D.M., Delyzer, A.J., 1962. The fungus *empusa aphidis* hoffman parasitic on the woolly pine needle aphid, *schizolachnus pini-radiatae* (davidson). The Canadian Entomologist 94, 46–49. https://doi.org/10.4039/Ent9446-1
Gruber, F., Poprawski, T.J., Rey, E., 1991. Survey for natural enemies of *diuraphis noxia* (mordvilko) in eurasia. International Organisation for Biological and Integrated Control/West Palaearctic Regional Section Bulletin 14, 102–109.
Gruppe, A., Roemer, P., 1988. The lupin aphid (*macrosiphum albifrons* essig, 1911) (hom., aphididae) in west germany: Its occurrence, host plants and natural enemies. Journal of Applied Entomology 106, 135–143. https://doi.org/10.1111/j.1439-0418.1988.tb00576.x
Guesmi, J., Ben Halima-Kamel, M., Boughalleb, N., 2010. First report of aphidopathogenic fungi *fusarium semitectum* (berkeley and ravenel, 1875) and *fusarium sacchari* (butler and hafiz khan) gams (1971) on *capitophorus elaeagni* (del guercio) (hemiptera: aphididae). African Journal of Agricultural Research 5, 290–293.
Guesmi-Jouini, J., Karous, O., Boughalleb-M'hamdi, N., Ben Halima-Kamel, M., 2013. Monitoring and identification of causal agents of natural mycosis on *capitophorus elaeagni*, del guercio (hemiptera: Aphididae) in tunisian organic artichoke crop, in: Crop Protection Management in Mediterranean Organic Agriculture. Sousse, Tunisia.

Gustafsson, M., 1969. On species of the genus *entomophthora* fres. In sweden. III. Possibility of usage in biological control. Lantbrukshögskolans Annaler 35, 235–274.

Gustafsson, M., 1965. On species of the genus *entomophthora* fres. In sweden. I. Classification and distribution. Lantbrukshögskolans Annaler 31, 103–212.

Hajek, A.E., Scott, K.L., Sanchez-Peña, S.R., Tkaczuk, C., Lovett, B., Bushley, K.E., 2025. Annotated checklist of arthropod-pathogenic species in the entomophthoromycotina (fungi, zoopagomycota) in north america. MycoKeys 114, 329–366. https://doi.org/10.3897/mycokeys.114.139257

Hall, D.G., 1987. The sugarcane aphid, *melanaphis sacchari* (zehntner), in florida. Journal of the American Society of Sugar Cane Technologists 7, 26–29.

Hall, I.M., 1973. Pathogens of aphids, in: Lowe, A.D. (Ed.), Perspectives in Aphid Biology. The Entomological Society of New Zealand, Auckland, pp. 30–39.

Hall, I.M., Dietrick, E.J., 1955. Fungi on spotted alfalfa aphid. California Agriculture 9, 5, 16.

Hall, I.M., Dunn, P.H., 1957. Entomophthorous fungi parasitic on the spotted alfalfa aphid. Hilgardia 27, 159–181. https://doi.org/10.3733/hilg.v27n04p159

Hall, I.M., Lowe, A.D., Given, B.B., 1976. New records of aphid hosts of *entomophthora aphidis* and *e. Planchoniana* in new zealand. New Zealand Journal of Zoology 3, 111–112. https://doi.org/10.1080/03014223.1976.9517907

Hall, I.M., Lowe, A.D., Given, B.B., Oliver, E.H.A., 1979. Fungi attacking the blue-green lucerne aphid in new zealand. New Zealand Journal of Zoology 6, 473–474. https://doi.org/10.1080/03014223.1979.10428388

Hall, R.A., 1976. *Verticillium lecanii* on the aphid, *macrosiphoniella sanborni*. Journal of Invertebrate Pathology 28, 389–391. https://doi.org/10.1016/0022-2011(76)90016-1

Harper, A.M., 1958. Notes on behaviour of *pemphigus betae* doane (homoptera: Aphididae) infected with *entomophthora aphidis* hoffm. The Canadian Entomologist 90, 439–440. https://doi.org/10.4039/Ent90439-7

Harris, M.R., 1948. A phycomycete parasitic on aphids. Phytopathology 38, 118–122.

Hatting, J.L., 2002. Fungal parasitism of cereal aphids in south africa (PhD thesis). University of Natal, Pietermaritzburg, South Africa.

Hatting, J.L., Humber, R.A., Poprawski, T.J., Miller, R.M., 1999. A survey of fungal pathogens of aphids from south africa, with special reference to cereal aphids. Biological Control 16, 1–12. https://doi.org/10.1006/bcon.1999.0731

Henderson, N.C., 1977. Aspects of the biology and ecology of *acyrthosiphon kondoi* shinji (homoptera: aphididae) (Master's thesis). Massey University, Palmerston North, New Zealand.

Herlinda, S., Irsan, C., Mayasari, R., Septariani, S., 2010. Identification and selection of entomopathogenic fungi as biocontrol agents for *aphis gossypii* from south sumatra. Microbiology Indonesia 4, 137–142. https://doi.org/10.5454/mi.4.3.7

Hernández-Torres, I., López-Arroyo, J.I., Berlanga-Padilla, A.M., Loera-Gallardo, J., Acosta-Díaz, E., 2006. Efectividad de hongos entomopatógenos y vehículos de aplicación para el control del pulgón café de los cítricos *toxoptera citricida* kirkaldy (homoptera: aphididae). Vedalia 13, 17–26.

Holdom, D.G., 1983. In vitro culture of the aphid pathogenic fungus *entomophthora planchoniana* cornu (zygomycetes: entomophthorales). Australian Journal of Entomology 22, 188. https://doi.org/10.1111/j.1440-6055.1983.tb01870.x

Hopper, K.R., Coutinot, D., Chen, K., Mercadier, G., Halbert, S., Kazmer, D.J., Miller, R.H., Pike, K.S., Tanigoshi, L.K., 1998. Exploration for natural enemies to control *diuraphis noxia* (homoptera: Aphididae) in the united states, in: Quissenbery, S.S., Peairs, F.B. (Eds.), A Reponse Model for an Introduced Pest - the Russian Wheat Aphid. Entomological Society of America, Lanham, Maryland, pp. 166–182.

Houghton, W., Phillips, W., 1886. On *aphis rumicis*, linn., as a pest on the mangel-wurzel crops in shropshire in the autumn of 1885, and on a fungus destructive of the same *aphis*. The Annals and Magazine of Natural History 18, 1–7.

Hountondji, F.C.C., Lomer, C.J., Hanna, R., Cherry, A.J., Dara, S.K., 2002. Field evaluation of brazilian isolates of *neozygites floridana* (entomophthorales: Neozygitaceae) for the microbial control of cassava green mite in benin, west africa. Biocontrol Science and Technology 12, 361–370. https://doi.org/10.1080/09583150220128149

Huang, Z.-H., Feng, M.-G., Chen, X.-X., Liu, S.-S., 2008. Pathogenic fungi and parasitoids of aphids

present in air captures of migratory alates in the low-latitude plateau of yunnan, china. Environmental Entomology 37, 1264–1271. https://doi.org/10.1093/ee/37.5.1264

Hughes, R.D., 1963. Population dynamics of the cabbage aphid, *brevicoryne brassicae* (l.). The Journal of Animal Ecology 32, 393–424. https://doi.org/10.2307/2600

Hughes, R.D., Bryce, M.A., 1984. Biological characterization of two biotypes of pea aphid, one susceptible and the other resistant to fungal pathogens, coexisting on lucerne in australia. Entomologia Experimentalis et Applicata 36, 225–229. https://doi.org/10.1111/j.1570-7458.1984.tb03432.x

Ibrahim, H., 2017. Biodiversity of entomopathogenic fungi naturally infecting cabbage aphid, *brevicoryne brassicae* l. Journal of Plant Protection and Pathology 8, 631–634. https://doi.org/10.21608/jppp.2017.46947

Ibrahim, H.Y.E., Salam, A.M., Abdel-Mogib, M., El-nagar, Salem, H.A., Nada, M.S.A., 2011. Survey of entomopathogenic fungi naturally infecting cowpea aphid, *aphis craccivora*. koch. Journal of Plant Protection and Pathology 2, 1063–1070.

Jackson, D.J., 1918. Notes on the aphides of ross-shire, with descriptions of two species new to science. The Scottish Naturalist 76, 81–91.

Jandricic, S.E., Filotas, M., Sanderson, J.P., Wraight, S.P., 2014. Pathogenicity of conidia-based preparations of entomopathogenic fungi against the greenhouse pest aphids *myzus persicae*, *aphis gossypii*, and *aulacorthum solani* (hemiptera: aphididae). Journal of Invertebrate Pathology 118, 34–46. https://doi.org/10.1016/j.jip.2014.02.003

Jankevica, L., 2004. Ecological associations between entomopathogenic fungi and pest insects recorded in latvia. Latvijas entomologs 41, 60–65.

Jankevica, L., Cudare, Z., 2003. Investigations on entomophthoralean fungi in latvia. International Organisation for Biological and Integrated Control/West Palaearctic Regional Section Bulletin 26, 69–72.

Jones, T.H., 1918. Miscellaneous truck-crop insects in louisiana. I.-insects injurious to the globe artichoke in louisiana. Bulletin of the United States Department of Agriculture 703, 1–5.

Jones, T.H., 1915. Aphides or plant-lice attacking sugar-cane in porto rico. Puerto Rico. Board of Commissioners of Agriculture Bulletin 11, 7–19.

Joshi, S., Viraktamath, C.A., 2004. The sugarcane woolly aphid, *ceratovacuna lanigera* zehntner (hemiptera: Aphididae): Its biology, pest status and control. Current Science 87, 307–316.

Kariluoto, K.T., 1982. New records of the genus *entomophthora* fres. Infesting insects in finland. Annales Entomologici Fennici 48, 49–50.

Katerere, Y., 1983. The fungus *entomophthora planchoniana* cornu (non thaxter) the pine needle aphid, *eulachnus rileyi* (williams) zimbabwe. The Commonwealth Forestry Review 62, 271–273.

Keller, S., 2007. Arthropod-pathogenic entomophthorales from switzerland. III. First additions. Sydowia 59, 75–113.

Keller, S., 2006. Entomophthorales attacking aphids with a description of two new species. Sydowia 58, 38–74.

Keller, S., 1997. The genus *neozygites* (zygomycetes, entomophthorales) with special reference to species found in tropical regions. Sydowia 49, 118–146.

Keller, S., 1991. Arthropod-pathogenic entomophthorales of switzerland. II. *Erynia*, *eryniopsis*, *neozygites*, *zoophthora* and *tarichium*. Sydowia 43, 39–122.

Keller, S., 1987b. Arthropod-pathogenic entomophthorales of switzerland. I. *Conidiobolus*, *entomophaga* and *entomophthora*. Sydowia 40, 122–167.

Keller, S., 1987a. Observations on the overwintering of *entomophthora planchoniana*. Journal of Invertebrate Pathology 50, 333–335. https://doi.org/10.1016/0022-2011(87)90103-0

Keller, S., GC, Y.D., 2007. Insect pathogenic entomophthorales from nepal and india. Mitteilungen der Schweizerischen Entomologischen Gesellschaft = Bulletin de la Société Entomologique Suisse = Journal of the Swiss Entomological Society 80, 211–215. https://doi.org/10.5169/SEALS-402946

Keller, S., Suter, H., 1980. Epizootiologische untersuchungen über das *entomophthora*-auftreten bei feldbaulich wichtigen blattlausarten. Acta Oecologica 1, 63–81.

Kenneth, R.G., 1977. *Entomophthora turbinata* sp. N., a fungal parasite of the peach trunk aphid, *pterochloroides persicae* (lachnidae). Mycotaxon 6, 381–390.

Kenneth, R., Olmert, I., 1975. Entomopathogenic fungi and their insect hosts in israel: additions. Israel Journal of Entomology 10, 105–112.

Kenneth, R., Wallis, G., Olmert, Y., Halperin, J., 1971. A list of entomogenout fungi of israel. Israel Journal of Agricultural Research 21, 63–66.

Kerns, D.L., Gaylor, M.J., 1993. Biotic control of cotton aphids (homoptera: Aphididae) in cotton influenced by two insecticides. Journal of Economic Entomology 86, 1824–1834. https://doi.org/10.1093/jee/86.6.1824

Khan, S., Guo, L., Shi, H., Mijit, M., Qiu, D., 2012. Bioassay and enzymatic comparison of six entomopathogenic fungal isolates for virulence or toxicity against green peach aphids *myzus persicae*. African Journal of Biotechnology 11, 14193–14203. https://doi.org/10.5897/AJB12.1592

Kish, L.P., Majchrowicz, I., Biever, K.D., 1994. Prevalence of natural fungal mortality of green peach aphid (homoptera: Aphididae) on potatoes and nonsolanaceous hosts in washington and idaho. Environmental Entomology 23, 1326–1330. https://doi.org/10.1093/ee/23.5.1326

Kishore, V.P., Chandra, S.V., Bhavani, B., Upendhar, S., 2019. *Cladosporium cladosporioides*: A new report of parasitism on sugarcane woolly aphid, *ceratovacuna lanigera* zehntner. Journal of Entomology and Zoology Studies 7, 1122–1126.

Kitazawa, K., Fujisawa, I., Imabayashi, S., 1984. [Isolation of *verticillium lecanii* (zimm.) viegas affecting aphids and greenhouse whitefly in japan]. Annals of the Phytopathological Society of Japan () 50, 574–581.

Kleespies, R.G., Huger, A.M., Zimmermann, G., 2010. Database on diagnosis of arthropod diseases in the internet of the julius kühn-institute. Journal of Applied Entomology 134, 80–80. https://doi.org/10.1111/j.1439-0418.2009.01431.x

Klingen, I., Salinas, S.H., Meadow, R., 2002. Checklist of naturally occurring pathogens of insects and mites in norway. Norwegian Journal of Entomology 49, 23–28.

Kobayashi, Y., Mogami, K., Aoki, J., 1984. Ultrastructural studies on the hyphal growth of *erynia neoaphidis* in the green peach aphid, *myzus persicae*. Transactions of the Mycological Society of Japan 25, 425–434.

Koval, E.Z., 1974. Identification guide to entomophilous fungi of the USSR. Naukova dumka, Kiev, Ukraine.

Krejzová, R., 1987. Pathogenicity of *erynia neoaphidis* for *acyrthosiphon pisum*. Ceská Mykologie 41, 65–72.

Krejzová, R., 1976. Record of *entomophthora* (*triplosporium*) *fresenii* (nowakowski) gustafsson in czechoslovakia and its taxonomic evaluation. Ceská Mykologie 30, 207–213.

Krejzová, R., 1974. List of species and strains of the genus *entomophthora* cultivated in the collextion of the institute of entomology. Ceská Mykologie 28, 189–191.

Krenner, J.A., 1935. Néhány adat hazánk g'ombaflórájához / einige beiträge zur pilzflora ungarns. Botanikai Közlemények 32, 201–202.

Krzyzanowski, R., Tkaczuk, C., 2018. Wystepowanie grzybów entomopatogenicznych w populacjach mszycy rhopalosiphum padi (l.) zerujacej na pierwotnych i wtórnych roslinach zywicielskich. Progress in Plant Protection 58, 55–62. https://doi.org/10.14199/ppp-2018-007

Kuno, G., 1975. Preliminary survey of microorganisms associated with some insects in puerto rico. Journal of Agriculture of the University of Puerto Rico 59, 69–74.

Kuntz, W.A., 1928. Entomogenous fungi. Florida Agricultural Experiment Station Annual Report 77R.

Kuntz, W.A., 1927. Diseases of the citrus aphid. Florida Agricultural Experiment Station Annual Report 64R–67R.

Kuntz, W.A., 1926. Aphid diseases. Florida Agricultural Experiment Station Annual Report 84R.

Lagerheim, G., 1899. En svampepidemi på bladlöss sommaren 1896. Entomologisk Tidskrift 20, 127–132.

Latgé, J.P., 1975. Croissance et sporulation de 6 espèces d'entomophthorales 1. Influence de la nutrition carbonée. Entomophaga 20, 201–207. https://doi.org/10.1007/BF02371660

Latteur, G., 1977. Sur la possibilité d'infection directe d'aphides par entomophthora a partir de sols hébergeant un inoculum nature. Comptes Rendus Hebdomadaires des Séances de l'Acamémie des Sciences 284, 2253.

Lázzari, S.N., 1985. Inimigos naturais dos afídeos (homoptera, aphididae) da cevada (*hordeum* sp.) no paraná. Anais da Sociedade Entomológica do Brasil 14, 5–15.

Leatherdale, D., 1970. The arthropod hosts of entomogenous fungi in britain. Entomophaga 15, 419–435. https://doi.org/10.1007/BF02370311

Lee, M., Yoon, C.-S., Yun, T., Kim, H.-S., Yoo, J.-K., 2002. Selection of a highly virulent *verticillium lecanii* strain against *trialeurodes vaporariorum* at various temperatures. Journal of Microbiology and Biotechnology 12, 145–148.

Lefort, F., Fleury, D., Fleury, I., Coutant, C., Kuske, S., Kehrli, P., Maignet, P., 2014. Pathogenicity of entomopathogenic fungi to the green peach aphid *myzus persicae* sulzer (aphididae) and the european tarnished bug *lygus rugulipennis* poppius (miridae). Egyptian Journal of Biological Pest Control 24, 379–386.

Li, H., 1987. [Preliminary report on investigation of vegetable main pest pathogens]. [Natural Enemies of Insects] 9, 194–198.

Li, H., Kang, X., 1989. [A survey on the entomophthorales fungi on vegetable aphids in changsha area]. [Chinese Journal of Biological Control] 5, 82–83. https://doi.org/10.16409/j.cnki.2095-039x.1989.02.012

Li, W., Hè, Y., Wáng, J., Zhang, Z., Sòng, D., 2003. [Study on the resources of entomogenous fungi in pangquangou national nature reserve]. Forest Research 16, 339–342. https://doi.org/10.13275/j.cnki.lykxyj.2003.03.016

Li, W., Sheng, C.-F., 2007. Occurrence and distribution of entomophthoralean fungi infecting aphids in mainland china. Biocontrol Science and Technology 17, 433–439. https://doi.org/10.1080/09583150701213802

Li, W., Wáng, X., Li, Z., Xu, W., Shèng, C., 2004. [Survey of entomophthoralean fungi in shandong]. Entomological Knowledge () 41, 350–353.

Li, Z., 2000. [Entomophthorales]. Beijing: Science Press, Beijing.

Li, Z., 1986. [*Erynia anhuiensis* a new pathogen of aphids]. Acta Mycologica Sinica 5, 1–6. https://doi.org/10.13346/j.mycosystema.1986.01.001

Li, Z., Fan, M., Wang, B., Huang, B., 1999. Entomophthoralean fungi in china. Journal of Anhui Agricultural University () 26, 286–291.

Li, Z., Wáng, J., Lu, X., 1989. [Two entomophthoralean fungi causing extensive epizootics in forest and tea plants pests]. Acta Mycologica Sinica 8, 81–85. https://doi.org/10.13346/j.mycosystema.1989.02.001

Londoño Zuluaga, M.E., Tamayo Molano, P.J., 1983. *Erynia neoaphidis*, hongo que controla el áfido *macrosiphum euphorbiae* en arveja. ICA-Informa 27, 5–8.

Lopez Lastra, C.C., Hatting, J., 2010. Identification, isolation and virulence of entomopathogenic fungi collected from cereal aphids in argentina and south africa. Trabzon, Turkey, p. 81.

López Lastra, C.C., Scorsetti, A.C., 2007. Revisión de los hongos entomophthorales (zygomycota: Zygomycetes) patógenos de insectos de la república argentina. Boletín de la Sociedad Argentina de Botánica 42, 33–37.

Lowe, A.D., 1963. The fungus *entomophthora aphidis* hoffman parasitic on the cabbage aphid (*brevicoryne brassicae* l.) in new zealand. New Zealand Journal of Agricultural Research 6, 314–317. https://doi.org/10.1080/00288233.1963.10418140

Lowe, A.D., Hall, I.M., 1978. Additional records of *entomophthora aphidis* and *e. Planchoniana* from aphids in new zealand. New Zealand Journal of Zoology 5, 493–493. https://doi.org/10.1080/03014223.1978.10428333

Lpez Lastra, C.C., Scorsetti, A.C., 2006. Hongos patgenos de insectos en argentina (zygomycetes: entomophthorales). Revista de Biología Tropical 54, 311–315. https://doi.org/10.15517/rbt.v54i2.13871

Lpez-Lastra, C.C., Toledo, A.V., Manfrino, R.G., Gutierrez, A.C., 2019. Southernmost records of entomophtoromycotina. Updated review of entomophthoralean fungal insect pathogens of argentina. Caldasia 41, 349–357. https://doi.org/10.15446/caldasia.v41n2.72653

Lù, W., Wáng, M., 1988. [Investigation and identification of several aphids in shandong province]. [Microbiology] 15, 155–159. https://doi.org/10.13344/j.microbi ol.china.1988.04.005

Lù, W., Wáng, M., Zhào, J., 1988. [Primary study on the epizootics in aphids caused by entomophthorales]. [Research and Application of Entomogenous Fungi in China] 1, 168–171.

Luangsa-ard, J.J., Mongkolsamrit, S., Thanakitpipattana, D., Khonsanit, A., Tasanathai, K., Noisripoom, W., Humber, R.A., 2017. Clavicipitaceous entomopathogens: New species in *metarhizium* and a new genus *nigelia*. Mycological Progress 16, 369–391. https://doi.org/10.1007/s11557-017-1277-1

Lynch, A.M., 2014. Spruce aphid (*elatobium abietinum* walker) (hemiptera: aphididae), in: Van Driesche, R., Reardon, R. (Eds.), The Use of Classical Biological Control to Preserve Forests in North America. United States Department of Agriculture Forest Service, USA, pp. 259–270.

MacLeod, D.M., 1956. Notes on the genus *empusa* cohn. Canadian Journal of Botany 34, 16–26. https://doi.org/10.1139/b56-002

MacLeod, D.M., Müller-Kögler, E., 1973. Entomogenous fungi: *Entomophthora* species with pear-shaped to almost spherical conidia (entomophthorales: entomophthoraceae). Mycologia 65, 823–93. https:

//doi.org/10.2307/3758521
MacLeod, D.M., Müller-Kögler, E., Wilding, N., 1976. *Entomophthora* species with *e. Muscae*-like conidia. Mycologia 68, 1–29. https://doi.org/10.2307/3758894
MacLeod, D.M., Tyrrell, D., Soper, R.S., 1979. *Entomophthora canadensis* n. Sp., a fungus pathogenic on the woolly pine needle aphid, *schizolachnus piniradiatae*. Canadian Journal of Botany 57, 2663–2672. https://doi.org/10.1139/b79-316
Majchrowicz, I., Semczyszak, A., 1996. The effect of air humidity on the development of epizootics caused by entomophthorales in populations of *myzus persicae* (homoptera, aphididae). International Organisation for Biological and Integrated Control/West Palaearctic Regional Section Bulletin 19, 225–231.
Manfrino, R.G., 2014. Hongos entomophthorales patógenos de pulgones plaga de cultivos de cereales y hortícolas de la región pampeana de la argentina: Estudios comparativos de la diversidad y prevalencia (PhD thesis). Universidad Nacional de La Plata.
Manfrino, R.G., Castrillo, L.A., Lastra, C.C.L., Toledo, A.V., Ferrari, W., Jensen, A.B., 2020. Morphological and molecular characterization of entomophthorales (entomophthoromycota: Entomophthoromycotina) from argentina. Acta Mycologica 55. https://doi.org/10.5586/am.5522
Manfrino, R.G., Gutiérrez, A.C., Steinkraus, D.C., Salto, C.E., Lpez Lastra, C.C., 2014a. Prevalence of entomophthoralean fungi (entomophthoromycota) of aphids (hemiptera: Aphididae) on solanaceous crops in argentina. Journal of Invertebrate Pathology 121, 21–23. https://doi.org/10.1016/j.jip.2014.06.003
Manfrino, R.G., Hatting, J.L., Humber, R.A., Salto, C.E., Lpez Lastra, C.C., 2014b. Natural occurrence of entomophthoroid fungi (entomophthoromycota) of aphids (hemiptera: Aphididae) on cereal crops in argentina. Annals of Applied Biology 164, 151–158. https://doi.org/10.1111/aab.12089
Manfrino, R.G., Zumoffen, L., Salto, C.E., Lpez Lastra, C.C., 2014c. Natural occurrence of entomophthoroid fungi of aphid pests on *medicago sativa* l. In argentina. Revista Argentina de Microbiología 46, 49–52. https://doi.org/10.1016/S0325-7541(14)70048-3
Manfrino, R.G., Zumoffen, L., Salto, C.E., Lpez Lastra, C.C., 2013. Potential plant-aphid-fungal associations aiding conservation biological control of cereal aphids in argentina. International Journal of Pest Management 59, 314–318. https://doi.org/10.1080/09670874.2013.869372
Martínez Batista, E., 2009. Selección de hongos entomopatógenos para el control biológico de afidos (t. Citricida) de coclé (Master's thesis). Universidad de Panamá, Panama (city), Panamá.
Mathai, S., Geetha, D., Mohandas, N., 1998. Use of different locally available and cheaper substrates for mass multiplication of *fusarium pallidoroseum* (cooke) sacc. An effective fungal pathogen against the pea aphid *aphis craccivora* koch. New Trends in Biotechnology 249–251.
Mathai, S., Padmakumari, G., Nalinakumari, T., Hebsybai, 1990. *Mucor circinelloides* van tieghem, an entomogenous fungus of the banana aphid *pentalonia nigronervosa* coq. (aphididae:hemiptera). Journal of Biological Control 4, 124. https://doi.org/10.18311/jbc/1990/15315
Mattirolo, O., 1898. Sulla comparsa in italia della *entomophthora planchoniana* cornu, parassita degli afidi e sulla importanza die questi spezies per l'orticoltura e l'agricoltura. Le stazioni sperimentali agricole Italiane 31, 315–327.
Maxson, A.C., 1916. Some unpublished notes on *pemphigus betae* doane. Journal of economic entomology 9, 500–504.
Mdellel, L., Guesmi, J., Ben Halima-Kamel, M., 2015. Identification of two entomopathogenic fungi naturally infecting *pterochloroides persicae* (cholodkovsky 1899) (hemiptera, aphididae) in peach orchards in tunisia. European Journal of Environmental Sciences 5, 158–160. https://doi.org/10.14712/23361964.2015.90
Mehetre, S., Mukherjee, P., Kale, S., 2008. Natural occurrence of *gibberella fujikuroi* and its potential for control of the woolly aphid *ceratovacuna lanigera* (homoptera: Aphididae) in indian sugarcane plantations. Biocontrol Science and Technology 18, 215–220. https://doi.org/10.1080/09583150701790619
Messing, R.H., Aliniazee, M.T., 1985. Natural enemies of *myzocallis coryli* (hom.: Aphididae) in oregon hazelnut orchards. Journal of the Entomological Society of British Columbia 82, 14–18.
Michaud, J.P., Browning, H.W., 1999. Seasonal abundance of the brown citrus aphid, *toxoptera citricida*, (homoptera: Aphididae) and its natural enemies in puerto rico. The Florida Entomologist 82, 424–447. https://doi.org/10.2307/3496869
Miczulski, B., Abo Kaf, N., 1994. Patogeniczne grzyby znalezione na mszycach zbozowych w rejonie lublina. Wiadomosci Entomologiczne 13, 63.
Mietkiewski, R., Van der Geest, L.P.S., 1985. Notes on entomophthoraceous fungi infecting insects in the

netherlands. Entomologische Berichten 45, 190–192.

Milner, R.J., 1985. Distribution in time and space of resistance to the pathogenic fungus *erynia neoaphidis* in the pea aphid *acyrthosiphon pisum*. Entomologia Experimentalis et Applicata 37, 235–240. https://doi.org/10.1111/j.1570-7458.1985.tb03480.x

Milner, R.J., Bourne, J., 1983. Influence of temperature and duration of leaf wetness on infection of *acyrthosiphon kondoi* with *erynia neoaphidis*. Annals of Applied Biology 102, 19–27. https://doi.org/10.1111/j.1744-7348.1983.tb02662.x

Milner, R.J., Holdom, D.G., 1986. First record of *neozygites fresenii* (nowakowski) batko, a fungal pathogen of aphids, in australia. Australian Journal of Entomology 25, 85–86. https://doi.org/10.1111/j.1440-6055.1986.tb01079.x

Milner, R.J., Mahon, R.J., Brown, W.V., 1983. A taxonomic study of the *erynia neoaphidis* remaudière & hennebert (zygomycetes: Entomophthoraceae) group of insect pathogenic fungi, together with a description of the new speceies *erynia kondoiensis*. Australian Journal of Botany 31, 173. https://doi.org/10.1071/BT9830173

Milner, R.J., Soper, R.S., Lutton, G.G., 1982. Field release of an israelistrain of the fungus *zoophthora radicans* (brefeld) batko for biological control of *therioaphis trifolii* (monell) f. *Maculata*. Australian Journal of Entomology 21, 113–118. https://doi.org/10.1111/j.1440-6055.1982.tb01776.x

Milner, R.J., Teakle, R.E., Lutton, G.G., Dare, F.M., 1980. Pathogens (phycomycetes: Entomophthoraceae) of the blue-green aphid *acyrthosiphon kondoi* shinji and other aphids in australia. Australian Journal of Botany 28, 601. https://doi.org/10.1071/BT9800601

Missonnier, J., Robert, Y., Thoizon, G., 1970. Circonstances épidémiologiques semblant favoriser le développement des mycoses a entomophthorales chez trois aphides, *aphis fabae* scop., *capitophorus horni* börner et *myzus persicae* (sulz.). Entomophaga 15, 169–190. https://doi.org/10.1007/BF02371870

Mitina, G.V., Tokarev, Yu.S., Yli-Mattila, T., 2013. Species identification of entomopathogenic fungi of the genus *lecanicillium* by molecular markers. European Entomological Journal 12, 431–437.

Mokrzecki, Z., 1928. Sprawozdanie z dzialalnosci zakladu ochrony lasu i entomologji w skierniewicach. 1924-1927. The Polish Journal of Entomology 6, 265–325.

Montalva, C., 2008. Evaluación de la virulencia de dos cepas de ¡i¿lecanicillium lecanii¡/i¿ (zimm.) zare y gams para el control biológico de (¡i¿buckton¡/i¿) (Master's thesis). Facultad de Ciencias Forestales, Universidad Austral de Chile, Valdivia, Chile.

Montalva, C., Barta, M., Pérez, E.R., Flores, E.V., 2013. *Neozygites osornensis* sp. Nov., a fungal species causing mortality to the cypress aphid *cinara cupressi* in chile. Mycologia 105, 661–669. https://doi.org/10.3852/12-125

Montalva, C., Barta, M., Rojas, E., Gutiérrez, M., Valenzuela, E., 2014. *Neozygites* species associated with aphids in chile: Current status and new reports. Mycotaxon 129, 233–245. https://doi.org/10.5248/129.233

Montalva, C., Gutiérrez, M., Rojas, E., Lanfranco, D., Valenzuela, E., 2010a. Prospección de hongos entomopatógenos del pulgón del ciprés en dos regiones ecológicas de chile. i. Boletín Micológico 25, 1–7. https://doi.org/10.22370/bolmicol.2010.25.0.1274

Montalva, C., Luz, C., Humber, R.A., 2016. *Neozygites osornensis* (neozygitales: Neozygitaceae) infecting *cinara* sp. (Hemiptera: Aphididae) in brazil. Neotropical Entomology 45, 227–230. https://doi.org/10.1007/s13744-016-0369-y

Montalva, C., Rojas, E., Barta, M., Valenzuela, E., 2015. Observations on the occurrence of entomopathogenic fungi infecting tree-dwelling aphids in southern chile. Teresópolis, Rio de janeiro, Brazil.

Montalva, C., Rojas, E., Ruiz, C., Lanfranco, D., 2010b. El pulgn del ciprés en chile: Una revisin de la situacin actual y antecedentes del control biolgico. Bosque 31, 81–88. https://doi.org/10.4067/S0717-92002010000200001

Montalva, C., Zhou, X., Rojas, E., Arismendi, N., Wang, J.-H., 2025. Report of insect-pathogenic fungi from Zhejiang Province, Southeast China. Biologia. https://doi.org/10.1007/s11756-025-02021-3

Mosqueda Anaya, J.A., Landeros Jaime, F., Ramírez Baltazar, S., Santiago Basilio, M.Á., Vergara Pineda, S., Cervantes Chávez, J.A., Esquivel Naranjo, E.U., 2018. Hongos asociados a cadáveres de insectos plaga en el estado de querétaro, méxico. Scientia Fungorum 47, 25–35. https://doi.org/10.33885/sf.2018.47.1190

Moubasher, A.H., Abdel-Rahman, M.A.A., Abdel-Mallek, A.Y., Hammam, G.H.A., 2010. Biodiversity of

entomopathogenic fungi infecting wheat and cabbage aphids in assiut, egypt. Journal of Basic & Applied Mycology 1, 53–60.

Nagalingam, B., Jayaraj, S., 1986. First record of *fusarium semitectum* berk and rav as an entomophagous fungus. Current Science 55, 377–378.

Nielsen, C., Eilenberg, J., Dromph, K., 2001a. Entomophthorales on cereal aphids: Characterisation, growth, virulence, epizootiology and potiential for microbial control. Bekæmpelsesmiddelforskning fra Miljøstyrelsen 53, 1–75.

Nielsen, C., Eilenberg, J., Harding, S., Oddsdottir, E., Halldrsson, G., 2001b. Geographical distribution and host range of entomophthorales infecting the green spruce aphid *elatobium abietinum* walker in iceland. Journal of Invertebrate Pathology 78, 72–80. https://doi.org/10.1006/jipa.2001.5045

Nielsen, C., Hajek, A.E., 2005. Control of invasive soybean aphid, *aphis glycines* (hemiptera: Aphididae), populations by existing natural enemies in new york state, with emphasis on entomopathogenic fungi. Environmental Entomology 34, 1036–1047. https://doi.org/10.1093/ee/34.5.1036

Nolla, J.A.B., 1929. *Acrostalagmus aphidum* oud. And aphid control. The Journal of the Department of Agriculture of Puerto Rico 13, 59–72.

Nowakowski, L., 1883. Entomophthoreae. Przyczynek do znajomosci pasorzytnych grzybkow sprawiajacych pomor owadow. Pamietnik Wydzial Akademii Umiejetnosci w Krakowie 8, 153–183.

O'Brien, P.J., Stoetzel, M.B., Navasero, R.C., Graves, J.B., 1993. Field biology studies of the cotton aphid, *aphis gossypii* glover. Southwestern Entomologist 18, 25–35.

Oatman, E.R., Platner, G.R., 1972. An ecological study of aphids on strawberry in southern california. Environmental Entomology 1, 339–343. https://doi.org/10.1093/ee/1.3.339

Ohnesorge, B., 1959. Die massenvermehrung der sitkalaus in nordwestdeutschland. Forstarchiv : forstwissenschaftliche Fachzeitschrift 30, 73–78.

Olfert, O.O., Doane, J.F., Carl, K., Erlandson, M.A., Goettel, M.S., 2002. *Diuraphis noxia* (kurdjumov), russian wheat aphid (homoptera: aphididae), in: Mason, P.G., Huber, J.T. (Eds.), Biological Control Programmes in Canada, 1981 - 2000. CABI Pub, Oxon, UK New York, pp. 110–114.

Papierok, B., 1989. On the occurrence of entomophthorales (zygomycetes) in finland. I. Species attacking aphids (homoptera, aphididae). Annales Entomologici Fennici 55, 63–69.

Papierok, B., 1978. Obtention in vivo des azygospores d'*entomophthora thaxteriana* petch, champignon pathogène de pucerons (homoptères aphididae). Comptes Rendus de l'Académie des Sciences 286, 1503–1506.

Papierok, B., Coremans-Pelseneer, J., 1980. Contribution à l'étude de *conidiobolus osmodes* drechsler (zygomycetes, entomophthoraceae), agent occasionnel d'épizooties chez les pucerons (homoptères, aphididae). Cryptogamie-Mycologie 1, 111–117. https://doi.org/10.5962/p.354054

Papierok, B., Dedryver, C.-A., Hullé, M., 2016. First records of aphid-pathogenic entomophthorales in the sub-antarctic archipelagos of crozet and kerguelen. Polar Research 35, 28765. https://doi.org/10.3402/polar.v35.28765

Papierok, B., Havukkala, I., 1986. Entomophthoraceous fungi parasitizing cereal aphids in finland. Annales entomologici Fennici 52, 36–38.

Park, H.-S., Chung, H.-G., Cho, Y.-J., Kim, S.-H., Kim, H.-H., Kim, J.-S., 2004. [Insect pests and natural enemies of *hibiscus syriacus* in korea]. The Korean Journal of Soil Zoology () 9, 1–5.

Patch, E.M., 1907. The potato plant louse. Maine Agricultural Experiment Station Technical Bulletin 147, 235–257.

Pavlyushin, V.A., 1998. [Scientific basis for the use of entomopathogens and antagonist microbes in phytosanitary optimization of greenhouse agrobiocenoses] (PhD thesis). All-Russian institute of plant protection (FSBSI VIZR), Saint Petersburg-Pushki, Russia.

Penteado, S.R.C., Reis-Filho, W., Iede, E.T., Grigoletti-Junior, A., Queirs, E.C., 2001. Ocorrência de *verticillium lecanii* em populações de *cinara pinivora* e *cinara atlantica*, no brasil, in: VII Simpsio de Controle Biolgico; Poços de Caldas, MG - Brasil. p. 324.

Pérez Consuegra, N., 2004. Manejo ecológico de plagas. Centro de Estudios de Desarrollo Agrario y Rural-CEDAR, Ciudad de La Habana, Cuba.

Petch, T., 1948. A revised list of british entomogenous fungi. Transactions of the British Mycological Society 31, 286–304. https://doi.org/10.1016/S0007-1536(48)80014-8

Petch, T., 1939. Notes on entomogenous fungi. Transactions of the British Mycological Society 23, 127–148.

https://doi.org/10.1016/S0007-1536(39)80019-5
Petch, T., 1936. New and rare yorkshire fungi. The Naturalist 950, 57–60.
Petch, T., 1932a. Notes on entomogenous fungi. Transactions of the British Mycological Society 16, 209–245. https://doi.org/10.1016/S0007-1536(32)80001-X
Petch, T., 1932b. A list of the entomogenous fungi of great britain. Transactions of the British Mycological Society 17, 170–178. https://doi.org/10.1016/S0007-1536(32)80012-4
Petch, T., 1931. Notes on entomogenous fungi. Transactions of the British Mycological Society 16, 55–75.
Pfrommer, W., Mendgen, K., 1992. Bekämpfung der mehligen kohlblattlaus (*brevicoryne brassicae*) mit dem entomopathogenen pilz *verticillium lecanii* im labor und freiland. Zeitschrift für Pflanzenkrankheiten und Pflanzenschutz 99, 209–217.
Piatkowski, J., Krzyzewska, A., 2007. Influence of some physical factors on the growth and sporulation of entomopathogenic fungi. Acta Mycologica 42, 255–265. https://doi.org/10.5586/am.2007.029
Pickering, J., Dutcher, J.D., Ekbom, B.S., 1989. An epizootic caused by *erynia neoaphidis* and *e. Radicans* (zygomycetes, entomophthoraceae) on *acyrthosiphon pisum* (horn., aphididae) on legumes under overhead irrigation. Journal of Applied Entomology 107, 331–333. https://doi.org/10.1111/j.1439-0418.1989.tb00265.x
Pickering, J., Gutierrez, A.P., 1991. Differential impact of the pathogen *pandora neoaphidis* (r.&h.) humber (zygomycetes: Entomophthorales) on the species composition of *acyrthosiphon* aphids in alfalfa. The Canadian Entomologist 123, 315–320. https://doi.org/10.4039/Ent123315-2
Poelen, J.H., Simons, J.D., Mungall, C.J., 2014. Global biotic interactions: An open infrastructure to share and analyze species-interaction datasets. Ecological Informatics 24, 148–159. https://doi.org/10.1016/j.ecoinf.2014.08.005
Poprawski, T.J., Parker, P.E., Tsai, J.H., 1999. Laboratory and field evaluation of hyphomycete insect pathogenic fungi for control of brown citrus aphid (homoptera: aphididae). Environmental Entomology 28, 315–321. https://doi.org/10.1093/ee/28.2.315
Poprawski, T.J., Wraight, S.P., 1998. Fungal pathogens of the russian wheat aphid (homoptera: aphididae), in: Quisenberry, S.S., Peairs, F.B. (Eds.), Response Model for an Introduced Pest - the Russian Wheat Aphid. Entomological Society of America, Lanham, Md., pp. 209–233.
Prado C., E., 1991. Artrópodos y sus enemigos naturales asociados a plantas cultivadas en chile. Título clave Boletín técnico - Instituto de Investigaciones Agropecuarias 169.
Rachappa, V.H., Lingappa, S., Patil, R.K., 2007. Occurrence of entomopathogenic fungi in northern karnataka. Journal of Ecobiology 20, 85–91.
Raj, D., Lakhanpal, G.C., 1998. Prevalence and impact of entomogenous fungus, *entomophthora aphidis* hoffmann on three important aphid species infesting rapeseed in the palam valley of himachal pradesh (india). Journal of Entomological Research 22, 73–76.
Ramaseshiah, G., 1968. *Entomophthora fresenii*, parasitic on aphids in india. Journal of Invertebrate Pathology 10, 439–441. https://doi.org/10.1016/0022-2011(68)90110-9
Ramaseshiah, G., 1967. The fungus *entomophthora coronata* parasitic on three species of aphids infesting crucifers in india. Journal of Invertebrate Pathology 9, 128–130. https://doi.org/10.1016/0022-2011(67)90053-5
Rao, V.P., 1968. Survey for natural enemies of aphids in india. Commonwealth Institute of Biological Control, Indian Station.
Raza, M.F., Yao, Z., Dong, X., Cai, Z., Zhang, H., 2017. Citrus insect pests and their non chemical control in china. Citrus Research & Technology 38, 122–138. https://doi.org/10.4322/crt.ICC117
Remaudière, G., 1977. Sur quelques aphidoidea de la polynésie française [hom.]. Bulletin de la Société Entomologique de France 82, 150–155.
Remaudière, G., Autrique, A., 1985. Contribution à l'ecologie des aphides africains. Etude FAO Production Végétale et Protection des PIantes 64, 1–214.
Remaudière, G., Hennebert, G.L., 1980. Révision systématique de *entomophthora aphidis* hoffm. In fres. Description de deux nouveaux pathogènes d'aphides. Mycotaxon 11, 269–321.
Remaudière, G., Keller, S., Papierok, B., Latgé, J.P., 1976a. Considération systématiques et biologiques sur quelques espèces d'*entomophthora* du groupe sphaerosperma pathogènes d'insectes (phycomycètes: entomophthoraceae). Entomophaga 21, 163–177.
Remaudière, G., Latgé, J.P., 1985. Importancia de los hongos patgenos de insectos (especialmente aphididae

y cercopidae) en méjico y perspectivas de uso. Boletín de sanidad vegetal. Plagas 11, 217–225.
Remaudière, G., Latgé, J.P., Michel, M.-F., 1981. Ecologie comparée des entomophthoracées pathogènes de pucerons en france littorale et continentale. Entomophaga 26, 157–178. https://doi.org/10.1007/BF02375030
Remaudière, G., Latgé, J.P., Smirnoff, W.A., 1978. Considérations ecologiques sur quelques entomophthorales pathogènes d'aphides communes dans l'est des u.s.a. Et du canada. Phytoprotection 59, 150–156.
Remaudière, G., Papierok, B., Latge, J.P., 1976b. La systématique et la notion de spécificité chez les aphides, chez leurs hyménoptères parasites et chez leurs champignons pathogènes. Médecine et Maladies Infectieuses 6, 418–423. https://doi.org/10.1016/S0399-077X(76)80129-1
Rèn, Yànfei, Wu, K., Gong, D., Huáng, Z., 2017. [Investigation on fungal pathogens of aphids and its occurrence in xiamen]. Subtropical Plant Science () 46, 43–47. https://doi.org/10.3969/j.issn.1009-7791.2017.01.008
Robert, Y., Rabasse, J.-M., Rouze-Jouan, J., 1972. Régulation naturelle des populations de *capitophorus horni* borner [hom: Aphididae] par hyménoptères aphidiidae et entomophthorales. Entomophaga 17, 59–69. https://doi.org/10.1007/BF02371073
Robert, Y., Rabasse, J.-M., Scheltes, P., 1973. Facteurs de limitation des populations d'*aphis fabae* scop. Dans l'ouest de la france. Entomophaga 18, 61–75. https://doi.org/10.1007/BF02373014
Rockwood, L.P., 1950. Entomogenous fungi of the family entomophthoraceae in the pacific northwest. Journal of Economic Entomology 43, 704–707. https://doi.org/10.1093/jee/43.5.704
Romero, M.G.Y. de, 1985. *Verticillium lecanii* (zimm.) viégas, en áfidos de la provincia de tucumán, argentina. CIRPON - Revista de Investigatión 3, 63–66.
Rondón, A., Arnal, E., Godoy, F., 1983. Comportamiento del *verticillium lecanii* (zimm.) viegas, patogeno del afido *toxoptera citricidus* (kirk.) en fincas citricolas de venezuela. Agronomía Tropical 30, 201–212.
Rong, I.H., Grobbelaar, E., 1998. South african records of associations between fungi and arthropods. African Plant Protection 4, 43–63.
Rostrup, O., 1935. Bidrag til danmarks svampeflora II. Dansk Botaisk Arkiv 8, 1–74.
Rostrup, O., 1916. Bidrag til danmarks svampeflora i. Dansk Botaisk Arkiv 2, 1–56.
Rozsypal, 1927. *Entomophthora aphidis* hoffm. Práce Moravské Prirodovédecké Spolecnosti 4, 353–364.
Rudraiah, M.P., Usman, S., 1955. Anepizootic of the oleander aphids. Science and Culture 21, 158–159.
Ruszkiewicz-Michalska, M., Balazy, S., Chelkowski, J., Dynowska, M., Pawlowska, J., Sucharzewska, E., Szkodzik, J., Tkaczuk, C., Wilk, M., Wrzosek, M., 2015. Preliminary studies of fungi in the biebrza national park (NE poland). Part III. Micromycetes - new data. Acta Mycologica 50, 1067. https://doi.org/0.5586/am.1067
Sabukwikopa, J., Muyango, S., 1991. Current status of *cinara cupressi* in burundi, its impact on forest resources and control operations, in: Exotic Aphid Pests of Conifers: A Crisis in African Forestry. FAO (Food; Agriculture Organization), Kenya, Africa.
Sahni, M.L., 1968. A preliminary report of an entomogenous fungus, *entomophthora exitialis*, on the wheat aphid, *rhopalosiphum maidis*. Journal of Invertebrate Pathology 10, 425–426. https://doi.org/10.1016/0022-2011(68)90102-X
Said, A.E., Nasruddin, A., 2017. Pathogenicity of several fungal entomopathogenic species against *aphis glycines* (homoptera: Aphididae) in south sulawesi, indonesia. Research Journal of Pharmaceutical, Biological and Chemical Sciences 8, 1084.
Samways, M.J., 1983. Interrelationship between an entomogenous fungus and two ant-homopteran (hymenoptera: Formicidae-hemiptera: Pseudococcidae & aphididae) mutualisms on guava trees. Bulletin of Entomological Research 73, 321–331. https://doi.org/10.1017/S0007485300008919
Sánchez, S.E.M., Santiago Álvarez, C., 1994. Nota sobre hongos entomoftorales de españa. Boletín de sanidad vegetal. Plagas 20, 517–520.
Sanchez-Peña, S.R., 2000. Entomopathogens from two chihuahuan desert localities in mexico. BioControl 45, 63–78. https://doi.org/10.1023/A:1009915308907
Sánchez-Peña, S.R., 1993. Entomogenous fungi associated with the cotton aphid in the texas high plains. Southwestern Entomologist 18, 69–71.
Santiago-Alvarez, C., 1991. The knowledge of fungal entomopathogens in spain. International Organisation for Biological and Integrated Control/West Palaearctic Regional Section Bulletin 14, 85–88.
Santos, A.C. da S., Diniz, A.G., Tiago, P.V., Oliveira, N.T. de, 2020. Entomopathogenic *fusarium* species: A

review of their potential for the biological control of insects, implications and prospects. Fungal Biology Reviews 34, 41–57. https://doi.org/10.1016/j.fbr.2019.12.002

Saranya, S., 2009. Biocontrol of cowpea aphid *aphis craccivora* (koch) using entomopathogenic fungi (Master's thesis). Kerala Agricultural University, Thrissur, Kerala, India.

Satyagopal, K., Sushil, S.N., Jeyakumar, P., Shankar, G., Sharma, O.P., Boina, D.R., Sain, S.K., Reddy, M.N., Rao, N.S., Sunanda, B.S., Asre, R., Kapoor, K.S., Arya, S., Kumar, S., Patni, C.S., Chattopadhyay, C., Badgujar, M.P., Choudhary, A.K., Varshney, S.K., Tippannavar, P.S., Basavraj, M.K., Thakare, A.Y., Halepyati, A.S., Patil, M.B., Sreenivas, A.G., 2014. AESA based IPM package for sugarcane. National Institute of Plant Health Management, Rajendranagar, Hyderabad.

Schander, 1912. Neue methoden zur bekämpfung des aaskäfers, des schildkäfers und der blattläuse. Zeitschrift des Vereins der Deutschen Zucker-Industrie 62, 8. https://doi.org/https://hdl.handle.net/2027/mdp.39015022733607

Schmitz, V., Dedryver, C.-A., Pierre, J.-S., 1993. Influence of an *erynia neoaphidis* infection on the relative rate of increase of the cereal aphid *sitobion avenae*. Journal of Invertebrate Pathology 61, 62–68. https://doi.org/10.1006/jipa.1993.1011

Schroeter, J., 1889. Entomophthorei, in: Cohn, F. (Ed.), Kryptogamenflora von Schlesien. Schlesische Gesellschaft für vaterländische Cultur, pp. 217–225.

Scorsetti, A.C., Humber, R.A., García, J.J., Lpez Lastra, C.C., 2007. Natural occurrence of entomopathogenic fungi (zygomycetes: Entomophthorales) of aphid (hemiptera: Aphididae) pests of horticultural crops in argentina. BioControl 52, 641–655. https://doi.org/10.1007/s10526-006-9045-1

Scorsetti, A.C., Pelizza, S.A., Cabello, M.N., 2012. New records of hypocrealean fungi infecting aphids and whiteflies: Pathogenicity against *myzus persicae* and interaction with its predator *eriopis connexa*. Biocontrol Science and Technology 22, 1099–1105. https://doi.org/10.1080/09583157.2012.710320

Serna-Domínguez, M.G., Andrade-Michel, G.Y., Rosas-Valdez, R., Castro-Félix, P., Arredondo-Bernal, H.C., Gallou, A., 2019. High genetic diversity of the entomopathogenic fungus *beauveria bassiana* in colima, mexico. Journal of Invertebrate Pathology 163, 67–74. https://doi.org/10.1016/j.jip.2019.03.007

Seskena, R., Petrova, V., Jankevica, M., Jankevica, L., 2008. Studies on ecological and physiological host range of entomopathogenic hyphomycetes. International Organisation for Biological and Integrated Control/West Palaearctic Regional Section Bulletin 31, 198–203.

Sewify, G.H., 1997. Occurrence and pathogenicity of entomopathogenic fungi in egypt. Ismailia, Suez Canal University, Egypt, pp. 380–395.

Seymour, A.B., 1929. Host index of the fungi of north america. Lehre : Cramer - Codicote/Herts. : Wheldon u. Wesley - New York : Stechert-Hafner.

Shah, P.A., Tymon, A., Pell, J.K., 2004. Novel strategies for aphid control using naturally occurring fungi. Home Grown Cereals Authority (HGCA), London.

Shands, W.A., Hall, I.M., Simpson, G.W., 1962. Entomophthoraceous fungi attacking the potato aphid in northeastern maine in 1960. Journal of Economic Entomology 55, 174–179. https://doi.org/10.1093/jee/55.2.174

Shands, W.A., Simpson, G.W., Hall, I.M., 1963. Importance of entomogenous fungi in controlling aphids on potatoes in northeastern maine. Maine Agricultural Experiment Station Technical Bulletin 6, 1–42.

Shands, W.A., Simpson, G.W., Hall, I.M., Gordon, C.C., 1972. Further evaluation of entomogenous fungi as a biological agent of aphid control in northeastern maine. Technical Bulletins of the Maine Agricultural and Forest Experiment Station 58.

Shands, W.A., Thompson, C.G., Simpson, G.W., Wave, H.E., 1958. Preliminary studies of entomogenous fungi for the control of potato-infesting aphids in maine. Journal of Economic Entomology 51, 184–186. https://doi.org/10.1093/jee/51.2.184

Shang, S.-W., Feng, M.-G., 2006. [Primary infection and contagious transmission of the entomophthorales at the early stage of *myzus persicae* mycosis]. Acta Ecologica Sinica () 26, 3380–3384.

Silvie, P., Delvare, G., Aberlenc, H.-P., Sognigbe, B., 1993. Contribution à l'inventaire faunistique du cotonnier au togo dans une optique de lutte intégrée. Coton et Fibres Tropicales 48, 313–325.

Silvie, P., Papierok, B., 1991. Les ennemis naturels d'insectes du cotonnier au tchad : Premières données sur les champignons de l'ordre des entomophthorales. Coton et Fibres Tropicales 46, 293–308.

Sivcev, I., 1991. Entomopathogenic fungi of cabbage aphid (*brevicoryne brassicae* l.). Plant Protection 42, 61–68.

Sletteng, C., 2014. Aphids and their natural enemies (hymenoptera and entomophthorales) in cereals - population dynamics, prevalence and effect of insect pathogenic fungi (Master's thesis). Norwegian University of Life Sciences, Ås, Norway.

Smirnoff, W.A., MacLeod, D.M., 1973. Une epizootie d'*entomophtora* sp. Dans une population du puceron du sapin (cinara curvipes) (hemiptera: aphididae). The Canadian Entomologist 105, 1369–1372. https://doi.org/10.4039/Ent1051369-10

Sòng, D., Sòng, S., Zhang, Z., Hè, Y., Wáng, J., Li, Y., 2001. [Study on the resources and pathogenicity of entomogenous fungi in lishan national nature reserve]. Forest Research 14, 628–632. https://doi.org/10.13275/j.cnki.lykxyj.2001.06.007

Sosa-Gmez, D.R., Lpez Lastra, C.C., Humber, R.A., 2010. An overview of arthropod-associated fungi from argentina and brazil. Mycopathologia 170, 61–76. https://doi.org/10.1007/s11046-010-9288-3

Sosa-Gomez, D.R., Humber, R.A., Moscardi, F., 2002. Entomopathogens associated with soybean/wheat production systems in brazil and argentina. Society for Invertebrate Pathology, p. 75.

Stary, P., Havelka, J., 1991. *Macrosiphum albifrons* essig, an invasive lupin aphid and its natural-enemy complex in czechoslovakia (homoptera, aphididae). Acta entomologica Bohemoslovaca 88, 111–120.

Steenberg, T., Eilenberg, J., 1995. Natural occurrence of entomopathogenic fungi on aphids at an agricultural field site. Czech Mycology 48, 89–96.

Steinhaus, E.A., Marsh, G.A., 1962. Report of diagnoses of diseased insects 1951-1961. Hilgardia 33, 349–490.

Stevenson, 1915. The entomogenous fungi of porto rico. Bulletin Porto Rico Agricultural Experiment Station, Insular Station, Río Piedras 10, 1–33.

Stevenson, J.A., 1918. A check list of porto rican fungi and a host index. Journal of Agriculture of the University of Puerto Rico 2, 125–264.

Störmer, K., Kleine, R., 1911. Über das verschwinden der blattläuse. Illustrierte landwirtschaftliche Zeitung 31, 557–558.

Su, D., Xin, J., 1963. [Notes on mycosis of two species of aphid in shanghai]. [Journal of Plant Protection] 2, 221–223. https://doi.org/10.13802/j.cnki.zwbhxb.1963.02.014

Sulc, K., 1910. Pseudovitellus und ähnliche bildungen der homopteren sind wohnstätten symbiotischer *saccharomyceten*. Königliche böhmische Gesellschaft der Wissenschaften.

Swirski, E., Amitai, S., 1999. Annotated list of aphids (aphidoidea) in israel. Israel Journal of Entomology 33, 1–120.

Thaxter, R., 1888. The entomophthoreae of the united states. Memoirs read before the Boston Society of Natural History 4, 133–201.

Thoizon, G., 1970. Spécificité du parasitisme des aphides par les entomophthorales. Annales de la Société Entomologique de France 6, 517–562.

Thoizon, G., 1967. *Entomophthora pyriformis*, sp. N., entomophthorale parasite de puceron. Entomophage 12, 303–307. https://doi.org/10.1007/BF02371434

Thomsen, D.A., Jensen, A.B., 2016. Molecular-genetic evidence of aphid pathogen *pandora kondoiensis* infection in *sitobion avenae* collected in denmark. Biocontrol Science and Technology 26, 414–418. https://doi.org/10.1080/09583157.2015.1110563

Thümen, F.K.A.E.J. von, 1877. Fungi nonnulli novi austriaci. Oesterreichische Botanische Zeitschrift 27, 12.

Tippannavar, P.S., Mallapur, C.P., Kulkarni, K.A., Patil, S.B., Yalmali, 2006. Record of a new entomopathogenic fungus on sugarcane woolly aphid. Current Science 91, 858.

Tkaczuk, C., Kontodimas, D.C., Martinou, A., Mantzoukas, S., Giannouri, D., 2009. [Isolation of entomopathogenic fungi from different habitats in greece using the *galleria* bait method, semiselective media and sampling cadavers]. pp. 251–255.

Tkaczuk, C., Mitkiewski, R., 2001. The growth of *hirsutella aphidis* petch - less-known pathogen of aphids - on media containing pesticides. Aphids and Other Homopterous Insects 8, 423–428.

Tymon, A.M., Shah, P.A., Pell, J.K., 2004. PCR-based molecular discrimination of *pandora neoaphidis* isolates from related entomopathogenic fungi and development of species-specific diagnostic primers. Mycological Research 108, 419–433. https://doi.org/10.1017/S0953756204009694

Ujjan, A.A., Shahzad, S., 2012. Use of entomopathogenic fungi for the control of mustard aphid (*lipaphis erysimi*) on canola (*brassica napus* l.). Pakistan Journal of Botany 44, 2081–2086.

Van Zwaluwenburg, R.H., 1917. Insects affecting coffee in porto rico. Journal of Economic Entomology 10, 513–517. https://doi.org/10.1093/jee/10.6.513

Villacarlos, L.T., 1995. Identification and culture of potential entomophthoralean species for insect pest control. NRCP-funded research project terminal report.

Villacarlos, L.T., Mejia, B.S., 2004. Philippine entomopathogenic fungi i. Occurrence and diversity. The Philippine Agricultural Scientist 87, 249–265.

Voronina, E.G., 1971. Entomophthorosis epizootics of the pea aphid *acyrthosiphon pisum* harris (homoptera, aphidoidea). Entomological Review 50, 444–453.

Vu, V.H., Hong, S.I., Kim, K., 2007. Selection of entomopathogenic fungi for aphid control. Journal of Bioscience and Bioengineering 104, 498–505. https://doi.org/10.1263/jbb.104.498

Wang, D., Deng, J., Pei, Y., Li, T., Jin, Z., Liang, L., Wang, W., Li, L., Dong, X., 2017. Identification and virulence characterization of entomopathogenic fungus *lecanicillium attenuatum* against the pea aphid *acyrthosiphon pisum* (hemiptera: aphididae). Applied Entomology and Zoology 52, 511–518. https://doi.org/10.1007/s13355-017-0503-2

Wáng, M., Lù, W., Chén, J., 1991. [Two new chinese records of *tarichium*]. [Research and Application of Entomogenous Fungi in China] 75–77.

Wáng, M., Zhào, J., Lù, W., 1986. [A pathogenic fungus of aphids, *erynia nouryi*]. [Research and Application of Entomogenous Fungi in China] 153–155.

Wang, Huang, 1988. A new record of *conidiobolus*, the observation and identification of *entomophthora muscae* and *conidiobolus obscurus*. Journal of Beijing Forestry University 10, 79–81.

Wanjama, J.K., Arama, P.F., 1999. Occurrence of entomopathogenic fungi and aphilinid parasitoids infecting cereal aphids in kenya, in: Proceedings of the Tenth Regional Wheat Workshop for Eastern, Central and Southern Africa, 14-18 September 1998. University of Stellenbosch, South Africa, pp. 404–407.

Waterhouse, D.F., Sands, D.P.A., 2001. Classical biological control of arthropods in australia. Australian Centre for International Agricultural Research.

Westrum, K., Klingen, I., 2007. Insect pathogenic fungi found in rosy apple aphid (*dysaphis plantaginea*) and green apple aphid (*aphis pomi*) in norway. International Organisation for Biological and Integrated Control/West Palaearctic Regional Section Bulletin 30, 147–149.

Westrum, K., Klingen, I., 2004. Insect pathogenic fungi and parasitoids as natural control agents of the apple aphids *aphis pomi* and *dysaphis plantaginea*, in: SIP 2004 37th Annual Meeting of the Society for Invertebrate Pathology, 7th International Conference on Bacillus Thuringiensis, Helsinki, Finland 1-6 August 2004 Book of Abstracts. Helsingin yliopisto, Helsinki, Finland, p. 67.

Wilding, N., Perry, J.N., 1980. Studies on *entomophthora* in populations of *aphis fabae* on field beans. Annals of Applied Biology 94, 367–378. https://doi.org/10.1111/j.1744-7348.1980.tb03952.x

Witlaczil, E., 1885. *Neozygites aphidis*, eine neue gregarinide. Archiv für Mikroskopische Anatomie 24, 599–603. https://doi.org/10.1007/BF02960395

Woroniecka-Siemaszko, J., 1928. Spostrzezenia nad szkodnikami roslin uprawnych, wystepujacemi w woj. Lubelskiem i czesci kieleckiego w latach 1926 i 1927. Pamietnik Panstwowego Instytutu Naukowego Gospodarstwa Wiejskiego w Pulawach 9, 216–251.

Wu, J., Wáng, D., 1991. [A checklist of entomogenous fungi in north and northeast china], in: [Research and Application of Entomogenous Fungi in China]. Fuzhou, Fujian, China, pp. 203–210.

Wu, J., Wáng, D., 1984. [The isolation, identification, culture and hosts of *conidiobolus thromboides*]. Acta Mycologica Sinica 3, 145–148. https://doi.org/10.13346/j.mycosystema.1984.03.003

Wu, J., Wáng, D., 1983. [Identification of three species of genus *entomophthora* fres. On aphids in beijing in autumn]. [Journal of Beijing Forestry College] 5, 41–45. https://doi.org/10.13332/j.1000-1522.1983.03.004

Wu, K., Huáng, Z., Chén, S., 2014. [A new *zoophthora occidentalis* pathogen of *myzus persicae* in china]. Fujian Journal of Agricultural Sciences () 29, 673–677.

Yeo, H., 2000. Mycoinsecticides for aphid management: A biorational approach (PhD thesis). University of Nottingham, Sutton Bonington, Leicestershire, United Kingdom.

Yoon, C.-S., Lee, M.-H., Lee, S.-H., Yoo, J.-K., Lee, J.-O., 1999a. First record of the entomopathogenic fungus *neozygites fresenii* on the aphid in korea. The Korean Journal of Mycology () 27, 66–67.

Yoon, C.-S., Sung, G.-H., Choi, B.-R., Yoo, J.-K., Lee, J.-O., 1998a. The aphid-attacking fungus *pandora neoaphidis*; the first observation and its host range in korea. The Korean Journal of Mycology () 26,

407–410.
Yoon, C.-S., Sung, G.-H., Lee, Gun-Hyung, Park, H.-S., Lee, J.-O., 1998b. *Entomophthora planchoniana* cornu (zygomycetes: Entomophthoraceae), the first observed pathogen of the green peach aphid *myzus persicae* in korea. The Korean Journal of Mycology () 26, 403–406.
Yoon, C.-S., Sung, G.-H., Lee, S., Yun, T.-Y., Lee, J.-O., 1998c. *Zoophthora phalloides* batko (zygomycetes: Entomophthoraceae), a fungal parasite of the aphid *dactynotus* species in korea. The Korean Journal of Mycology () 26, 413–415.
Yoon, C.-S., Sung, G.-H., Park, H.-S., Yoo, J.-K., Lee, J.-O., 1999b. Two entomopathogenic *conidiobolus* species first observed on the aphids in korea. The Korean Journal of Mycology () 27, 63–65.
Yoon, C.-S., Yun, T.-Y., Lee, Goen-Hyoung, Yoo, J.-K., 1998d. First record of the entomopathogenic fungus *zoophthora radicans* on the green peach aphid *myzus persicae* in korea. The Korean Journal of Mycology () 26, 339–402.
Yothers, W.W., Cole, F.R., 1924. The citrus aphid (*aphis spiraecola* patch). Citrus Industry 5, 6–8, 18–19.
Zambrano-Gutiérrez, J., Alatorre-Rosas, R., Carrillo-Benítez, M.G., Lomelí-Flores, J.R., Guzmán-Plazola, R.A., Azuara-Domínguez, A., Terán-Vargas, A.P., 2019. Species diversity of entomopathogenic fungi infecting the sugarcane aphid *melanaphis sacchari* a recently introduced pest in mexico. Advances in Microbiology 09, 38–55. https://doi.org/10.4236/aim.2019.91004
Zawadneak, M.A.C., Pimentel, I.C., Robl, D., Dalzoto, P., Vicente, V., Sosa-Gómez, D.R., Porsani, M., Cuquel, F.L., 2015. *Paecilomyces niveus* stolk & samson, 1971 (ascomycota: Thermoascaceae) as a pathogen of *nasonovia ribisnigri* (mosley, 1841) (hemiptera, aphididae) in brazil. Brazilian Journal of Biology 75, 158–162. https://doi.org/10.1590/1519-6984.08014
Zhang, et al., 2006. The pathogenic mechanisms to *brevicoryne brassicae* of *conidiobolus obscurus*. Chinese Journal of Biological Control 22, 38–41.
Zhang, X., Jì, H., Zhang, X., Xiàng, M., 1983a. Application studies on entomophthora fresenii. I. Pathogenic fungi, hosts and distribution. [Shaanxi Journal of Agricultural Sciences] 35–36;50.
Zhang, X., Jì, H., Zhang, X., Zhang, M., Xiàng, M., 1983b. Studies on the utilization of cotton aphid epidemic aphid mold II. Research on occurrence and epidemic law. [Shaanxi Journal of Agricultural Sciences] 33–34; 11.
Zhang, X., Táng, G., Jì, H., Li, S., Xiàng, M., 1983c. -. Chinese Science Bulletin () -, 948–951. https://doi.org/10.1360/csb1983-28-15-948
Zhou, X., Montalva, C., Arismendi, N., Hong, F., 2017a. *Neozygites linanensis* sp. Nov., a fungal pathogen infecting bamboo aphids in southeast china. Mycotaxon 132, 305–315. https://doi.org/10.5248/132.305
Zhou, X., Montalva, C., Arismendi, N., Hong, F., 2017b. Neozygites linanensis sp. Nov., a fungal pathogen infecting bamboo aphids in southeast China. Mycotaxon 132, 305–315. https://doi.org/10.5248/132.305
Zimmermann, G., 2008. The entomopathogenic fungi *isaria farinosa* (formerly *paecilomyces farinosus*) and the *isaria fumosorosea* species complex (formerly *paecilomyces fumosoroseus*): Biology, ecology and use in biological control. Biocontrol Science and Technology 18, 865–901. https://doi.org/10.1080/09583150802471812
Zúñiga, S.E., 1970. El pulgón verde pálido de las gramíneas. Agro informativo. Servicio Agrícola y Ganadero 136.
Zúñiga, S.E., 1967. Lista preliminar de áfidos que atacon cultivos en chile, sus huéspedes z enemigos naturales. Agricultura Técnica (Chile) 27, 165–177.
Zweigbaumówna, Z., 1924. Grzyby okolic skierniewic (les champignons des environs de skierniewice). Acta Societatis Botanicorum Poloniae 2, 275–301. https://doi.org/10.5586/asbp.1924.021
